# Necrostatin-1 Supplementation to Islet Tissue Culture Enhances the In-Vitro Development and Graft Function of Young Porcine Islets

**DOI:** 10.3390/ijms22168367

**Published:** 2021-08-04

**Authors:** Hien Lau, Shiri Li, Nicole Corrales, Samuel Rodriguez, Mohammadreza Mohammadi, Michael Alexander, Paul de Vos, Jonathan RT Lakey

**Affiliations:** 1 Department of Surgery, University of California Irvine, Irvine, CA 92868, USA; hlau3@hs.uci.edu (H.L.); ncorrale@uci.edu (N.C.); samuemr1@hs.uci.edu (S.R.); michaela@hs.uci.edu (M.A.); 2 Weill Cornell Medical College, Cornell University, Ithaca, NY 14850, USA; shl4018@med.cornell.edu; 3 Sue and Bill Gross Stem Cell Research Center, Department of Materials Science and Engineering, University of California Irvine, Irvine, CA 92697, USA; rezamoha@uci.edu; 4 Department of Biomedical Engineering, University of California Irvine, Irvine, CA 92697, USA; 5 University Medical Center Groningen, Department of Pathology and Medical Biology, University of Groningen, 9713 GZ Groningen, The Netherlands; p.de.vos@umcg.nl

**Keywords:** diabetes, xenotransplantation, pig islets

## Abstract

Pre-weaned porcine islets (PPIs) represent an unlimited source for islet transplantation but are functionally immature. We previously showed that necrostatin-1 (Nec-1) immediately after islet isolation enhanced the in vitro development of PPIs. Here, we examined the impact of Nec-1 on the in vivo function of PPIs after transplantation in diabetic mice. PPIs were isolated from pancreata of 8–15-day-old, pre-weaned pigs and cultured in media alone, or supplemented with Nec-1 (100 µM) on day 0 or on day 3 of culture (*n* = 5 for each group). On day 7, islet recovery, viability, oxygen consumption rate, insulin content, cellular composition, insulin secretion capacity, and transplant outcomes were evaluated. While islet viability and oxygen consumption rate remained high throughout 7-day tissue culture, Nec-1 supplementation on day 3 significantly improved islet recovery, insulin content, endocrine composition, GLUT2 expression, differentiation potential, proliferation capacity of endocrine cells, and insulin secretion. Adding Nec-1 on day 3 of tissue culture enhanced the islet recovery, proportion of delta cells, beta-cell differentiation and proliferation, and stimulation index. In vivo, this leads to shorter times to normoglycemia, better glycemic control, and higher circulating insulin. Our findings identify the novel time-dependent effects of Nec-1 supplementation on porcine islet quantity and quality prior to transplantation.

## 1. Introduction

The current standard treatment of Type 1 diabetes mellitus (T1DM) using subcutaneous administration of exogenous insulin is associated with daily stress and increased rate of severe hypoglycemic events due to suboptimal absorption and imprecise dosing [1]. To alleviate these adverse effects, islet allotransplantation using the Edmonton protocol has been deemed a propitious therapy to restore normoglycemia and promote long-term insulin independence in T1DM patients [2]. Despite these benefits, the worldwide implementation of islet transplantation has been hampered by the limited numbers of cadaveric donors and availability of high-quality human islets [3]. Thus, there is an urgent need to identify other sources of donor islets and maximize islet production.

In response to this shortage, porcine pancreata have been explored as a viable alternative because of their endless supply, easy husbandry, and similar physiology to human insulin [4]. Furthermore, advancements in genome editing and immunomodulation have made clinical islet xenotransplantation a potential replacement for human islet allotransplantation [5]. Islet isolation from young porcine pancreata, either neonatal (1–3 days old) or pre-weaned juvenile (8–15 days old), has been shown to be more scalable compared to the adult counterpart due to simpler isolation setup, less breeding time, and higher islet yield [6]. However, pro-longed in vitro tissue culture is required for the functional maturation of young porcine islets while significant islet loss has been reported over this essential culture period, making research efforts necessary to shorten the culture time, mitigate islet loss, and facilitate islet development [7,8].

Necrostatin-1 (Nec-1) acts as a specific small-molecule allosteric inhibitor of the receptor-interacting protein kinase 1 (RIPK1) [9]. The use of Nec-1 to inhibit necroptosis and reduce cell death has been studied in various cell lines and animal models of ischemic injury [10]. Recently, the effects of Nec-1 on islets have been explored after necroptosis was shown to play a key role in islet cell death [11]. The release of danger associated molecular patterns (DAMPs) from necroptotic islets can activate an inflammatory response to reduce islet viability and worsen graft outcomes [12]. After exposure to nitric oxide, Nec-1 treatment for 24 h decreased cell death and the release of DAMPs in murine islets [13]. Throughout 7-day tissue culture in hypoxic conditions, encapsulated human islets treated with Nec-1 maintained a stable amount of nuclear DNA and released less DAMPs [14]. Previously, we determined that culturing pre-weaned porcine islets (PPIs) in media supplemented with Nec-1 immediately after islet isolation for 7 days significantly augmented islet insulin content, proportion of endocrine cells, and in vitro insulin secretion [15]. Our recent studies have further shown that 100 μM of Nec-1 was the most effective dose to enhance the in vitro maturation of PPIs for up to 7 days during tissue culture [16,17]. However, these investigations lack a detailed comparison of the optimal timing to supplement Nec-1 to islet tissue culture and its impact on the in vivo function of PPIs. Thus, the present study compared the effects of supplementing Nec-1 on day 3 of tissue culture to immediately after islet isolation on PPIs in vitro and, for the first time, evaluated the in vivo function of PPIs treated with Nec-1 on day 3 of tissue culture after transplantation in streptozotocin-induced diabetic mice.

## 2. Results

### 2.1. Nec-1 Supplementation on Day 3 of Tissue Culture Improves Islet Recovery

**Figure 1:**
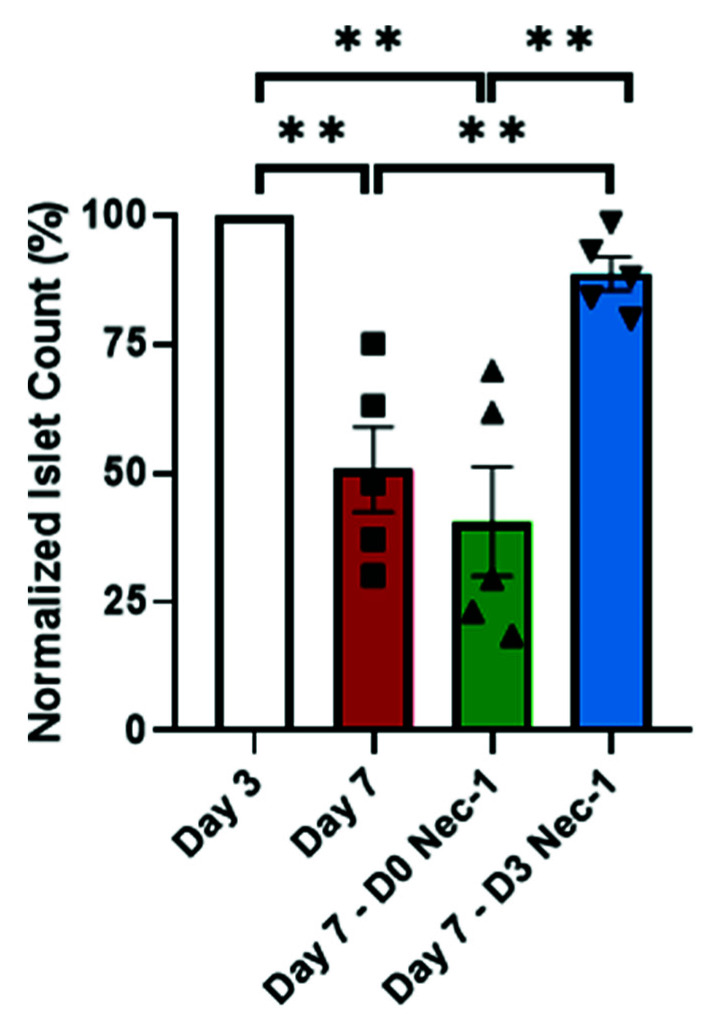
Islet recovery on day 7 of tissue culture in control media or media supplemented with Nec-1 either immediately after islet isolation (D0 Nec-1) or on day 3 of tissue culture (D3 Nec-1). Islet recovery was calculated as the percentage of IEQ per gram of pancreatic tissue normalized to day 3 of control untreated islets. *n* = 5 for each group. ** *p* < 0.01. Individual data points for islets cultured in control media (Day 7) or media supplemented with Nec-1 either immediately after islet isolation (D0 Nec-1) or on day 3 of tissue culture (D3 Nec-1) are shown within each bar graph as either squares, triangles, or inverted triangles, respectively. Data expressed as mean ± SEM.

On day 7, the number of recovered D3 Nec-1 treated islets was approximately twofold higher than untreated islets and D0 Nec-1 treated islets (Figure 1). In comparison to untreated islets on day 3, the islet loss was significantly higher in untreated and D0 Nec- 1 treated, but not D3 Nec-1 treated, islets on day 7 (Figure 1). However, Nec-1 supplementation either immediately after islet isolation or on day 3 did not affect islet viability compared to untreated islets on day 3 and day 7 (Supplementary Figure S1). As CalAM/PI staining alone might not have adequate sensitivity to detect the viability in the islet core, 7-AAD-stained dissociated islet cells was quantified via flow cytometry. Similarly, there was no significant difference in the viability between islet cells dissociated from untreated islets on day 3 and untreated-, D0 Nec-1 treated-, and D3 Nec-1 treated islets on day 7 (Supplementary Figure S2). Likewise, the OCR, a measurement of the fractional islet cell viability, was similar between untreated-, D0 Nec-1-, and D3 Nec-1 treated islets on both day 3 and 7 (Supplementary Figure S3) [18].

### 2.2. Nec-1 Supplementation on Day 3 of Tissue Culture Enhances the Development of Islet Endocrine Cells and Upregulates the Expression of GLUT2 in Beta Cells

**Figure 2:**
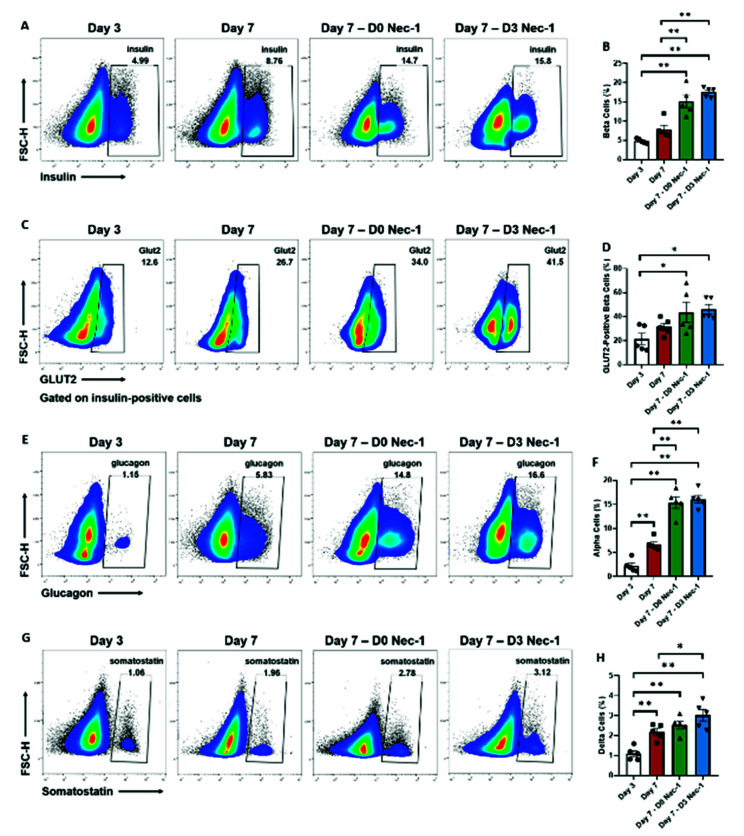
Flow cytometric analysis of cellular composition and GLUT2 expression in beta cells of PPIs on day 3 and 7 of tissue culture in control media or media supplemented with Nec-1 either immediately after islet isolation (D0 Nec-1) or on day 3 of tissue culture (D3 Nec-1). Islets were dissociated on day 3 and 7 of tissue culture using Accutase, stained with 7-AAD viability dye, anti-insulin, anti-glucagon, anti-somatostatin, and anti-GLUT2 antibodies, and analyzed by flow cytometry. (**A**) Representative flow cytometry plots of insulin staining of live islet cells on day 3 and 7 of tissue culture. (**B**) The percentage of insulin-positive beta cells on day 3 and 7 of tissue culture. (**C**) Representative flow cytometry plots of GLUT2 staining of live insulin-positive islet cells on day 3 and 7 of tissue culture. (**D**) The percentage of GLUT2-positive, insulin-positive beta cells on day 3 and 7 of tissue culture. (**E**) Representative flow cytometry plots of glucagon staining of live islet cells on day 3 and 7 of tissue culture. (**F**) The percentage of glucagon-positive alpha cells on day 3 and 7 of tissue culture. (**G**) Representative flow cytometry plots of somatostatin staining of live islet cells on day 3 and 7 of tissue culture. (**H**) The percentage of somatostatin-positive delta cells on day 3 and 7 of tissue culture. *n* = 5 for each group. * *p* < 0.05. ** *p* < 0.01. Individual data points for islets cultured in control media on day 3 (Day 3) or day 7 of tissue culture (Day 7) or in media supplemented with Nec-1 either immediately after islet isolation (D0 Nec-1) or on day 3 of tissue culture (D3 Nec-1) are shown within each bar graph as either circles, squares, triangles, or inverted triangles, respectively. Data expressed as mean ± SEM.

In comparison to untreated islets on day 3, the supplementation of Nec-1 to tissue culture media either immediately after islet isolation or on day 3 led to an approximately threefold increase in the beta-cell numbers on day 7 (Figure 2A,B). Moreover, the percentage of beta cells in D0 Nec-1- and D3 Nec-1 treated islets was twofold higher than untreated islets on day 7 (Figure 2A,B). D0 Nec-1- and D3 Nec-1 treated islets, but not untreated islets, also showed a twofold increase in the level of GLUT2-positive beta cells on day 7 compared to untreated islets on day 3 (Figure 2C,D).

Nec-1 supplementation either immediately after islet isolation or on day 3 resulted in a sevenfold and twofold increase in the level of alpha cells compared to untreated islets on day 3 and 7, respectively (Figure 2E,F). On day 7, the composition of delta cells increased by 2, 2.5, and 3 times for untreated, D0 Nec-1-, and D3 Nec-1 treated islets, respectively, compared to untreated islets on day 3 (Figure 2G,H). Furthermore, only D3 Nec-1 treated islets had a significantly higher level of delta cells than untreated islets on day 7 (Figure 2G,H).

### 2.3. Nec-1 Supplementation on Day 3 of Tissue Culture Improves the Differentiation of Pancreatic Progenitor Cells

**Figure 3:**
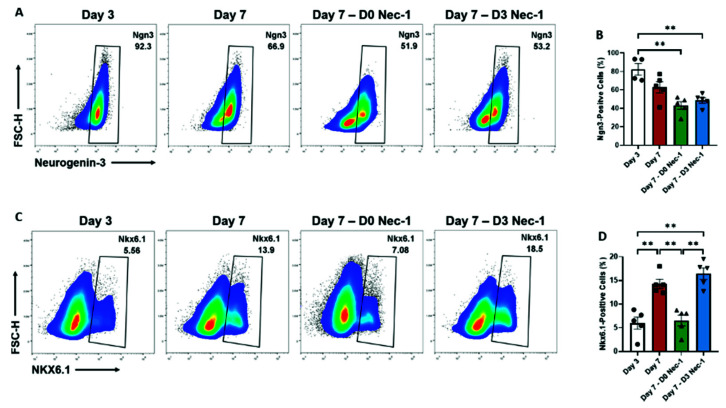
Flow cytometric analysis of endocrine cellular differentiation of PPIs on day 3 and 7 of tissue culture in control media or media supplemented with Nec-1 either immediately after islet isolation (D0 Nec-1) or on day 3 of tissue culture (D3 Nec-1). Islets were dissociated on day 3 and 7 of tissue culture using Accutase, stained with 7-AAD viability dye, anti-Ngn3, and anti-Nkx6.1 antibodies, and analyzed by flow cytometry. (**A**) Representative flow cytometry plots of neurogenin-3 staining of live islet cells on day 3 and 7 of tissue culture. (**B**) The percentage of Ngn3-positive cells on day 3 and 7 of tissue culture. (**C**) Representative flow cytometry plots of Nkx6.1 staining of live islet cells on day 3 and 7 of tissue culture. (**D**) The percentage of Nkx6.1-positive cells on day 3 and 7 of tissue culture. *n* = 5 for each group. ** *p* < 0.01. Individual data points for islets cultured in control media on day 3 (Day 3) or day 7 of tissue culture (Day 7) or in media supplemented with Nec-1 either immediately after islet isolation (D0 Nec-1) or on day 3 of tissue culture (D3 Nec-1) are shown within each bar graph as either circles, squares, triangles, or inverted triangles, respectively. Data expressed as mean ± SEM.

When compared to untreated islets on day 3, only islets treated with Nec-1 either immediately after islet isolation or on day 3, but not untreated islets, on day 7 had a significant decrease in the percentage of Ngn3-positive pancreatic progenitor cells (Figure 3A,B). In comparison to untreated islets on day 3, only untreated and D3 Nec-1 treated islets, but not D0 Nec-1 treated islets, on day 7 had a threefold increase in the percentage of Nkx6.1-positive pancreatic progenitor cells (Figure 3C,D). On day 7, the level of Nkx6.1-positive pancreatic progenitor cell in both untreated and D3 Nec-1 treated islets was significantly higher than D0 Nec-1 treated islets (Figure 3C,D).

### 2.4. Nec-1 Supplementation on Day 3 of Tissue Culture Stimulates the Proliferation of Islet Endocrine Cells

**Figure 4:**
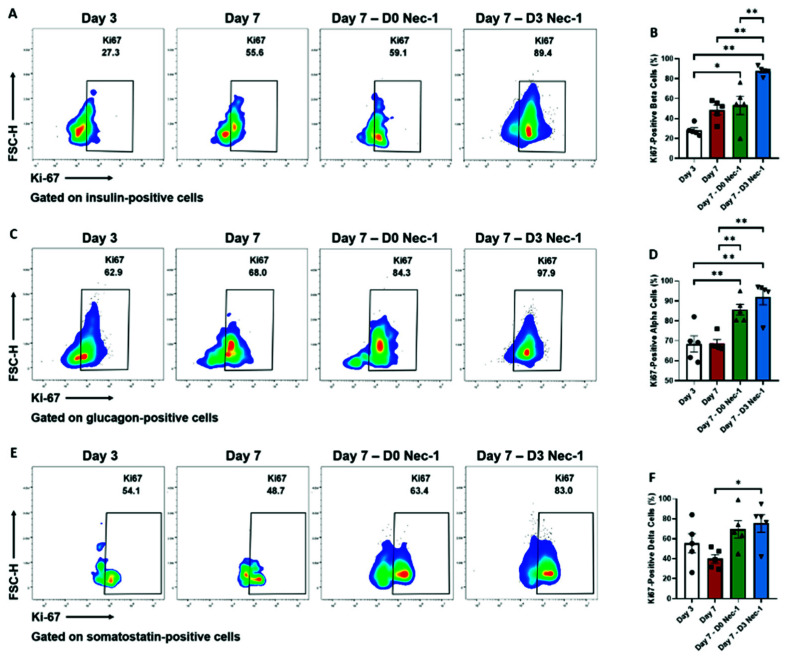
Flow cytometric analysis of proliferating endocrine cells of PPIs on day 3 and 7 of tissue culture in control media or media supplemented with Nec-1 either immediately after islet isolation (D0 Nec-1) or on day 3 of tissue culture (D3 Nec-1). Islets were dissociated on day 3 and 7 of tissue culture using Accutase, stained with 7-AAD viability dye, anti-Ki-67, anti-insulin, anti-glucagon, and anti-somatostatin antibodies, and analyzed by flow cytometry. (**A**) Representative flow cytometry plots of Ki-67 staining of live insulin-positive islet cells on day 3 and 7 of tissue culture. (**B**) The percentage of Ki-67-positive, insulin-positive beta cells on day 3 and 7 of tissue culture. (**C**) Representative flow cytometry plots of Ki-67 staining of live glucagon-positive islet cells on day 3 and 7 of tissue culture. (**D**) The percentage of Ki-67-positive, glucagon-positive alpha cells on day 3 and 7 of tissue culture. (**E**) Representative flow cytometry plots of Ki-67 staining of live somatostatin-positive islet cells on day 3 and 7 of tissue culture. (**F**) The percentage of Ki-67-positive, somatostatin-positive delta cells on day 3 and 7 of tissue culture. *n* = 5 for each group. * *p* < 0.05. ** *p* < 0.01.Individual data points for islets cultured in control media on day 3 (Day 3) or day 7 of tissue culture (Day 7) or in media supplemented with Nec-1 either immediately after islet isolation (D0 Nec-1) or on day 3 of tissue culture (D3 Nec-1) are shown within each bar graph as either circles, squares, triangles, or inverted triangles, respectively. Data expressed as mean ± SEM.

The number of Ki67-positive proliferating beta cells in islets treated with Nec-1 either immediately after islet isolation or on day 3, but not untreated islets, was significantly increased on day 7 compared to untreated islets on day 3 (Figure 4A,B). Strikingly, D3 Nec-1 treated islets had a twofold increase in the percentage of Ki67-positive proliferating beta cells compared to untreated and D0 Nec-1 treated islets on day 7 (Figure 4A,B).

While culturing islets in untreated media for 7 days did not enhance the percentage of Ki67-positive proliferating alpha cells, islets cultured in D0 Nec-1- or D3 Nec-1 treated had a significantly higher percentage of Ki67-positive proliferating alpha cells than untreated islets on day 3 (Figure 4C,D). The number of Ki67-positive proliferating alpha cells in D0 Nec-1- or D3 Nec-1 treated islets was also significantly increased compared to untreated islets on day 7 (Figure 4C,D). In addition, only D3 Nec-1 treated islets had a significantly higher percentage of Ki67-positive proliferating delta cells than untreated islets on day 7 (Figure 4E,F).

### 2.5. Nec-1 Supplementation on Day 3 of Tissue Culture Increases Insulin Content and Glucose-Stimulated Insulin Secretion

**Figure 5:**
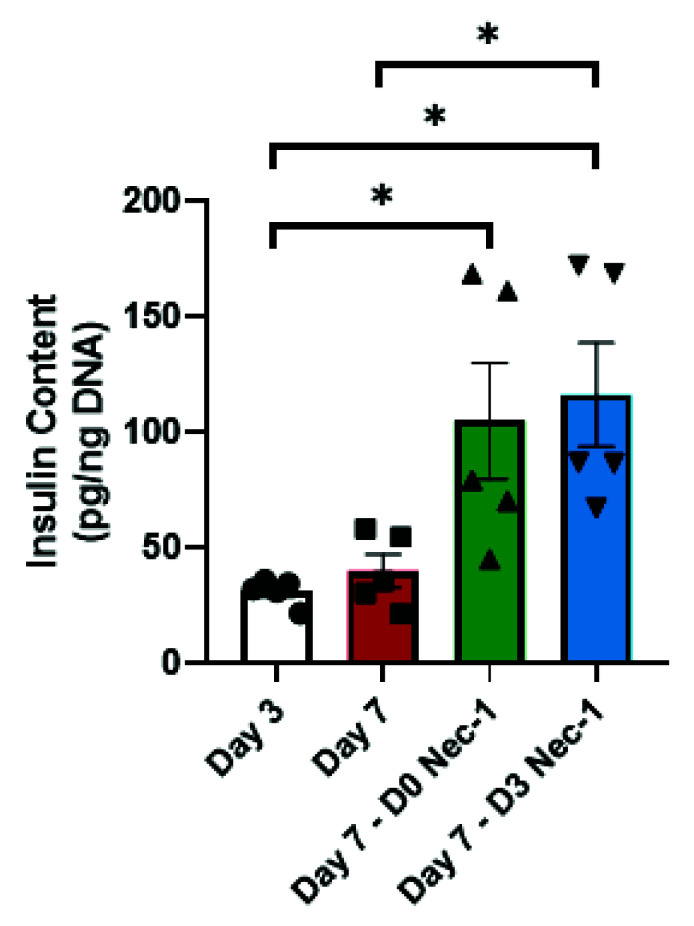
Insulin Content of PPIs on day 3 and 7 of tissue culture in control media or media supplemented with Nec-1 either immediately after islet isolation (D0 Nec-1) or on day 3 of tissue culture (D3 Nec-1). 150 IEQs per isolation was lysed, sonicated, and evaluated for insulin content on day 3 and 7 of tissue culture using standard porcine insulin ELISA. The amount of insulin was normalized to the sample DNA content. *n* = 5 for each group. * *p* < 0.05. Individual data points for islets cultured in control media on day 3 (Day 3) or day 7 of tissue culture (Day 7) or in media supplemented with Nec-1 either immediately after islet isolation (D0 Nec-1) or on day 3 of tissue culture (D3 Nec-1) are shown within each bar graph as either circles, squares, triangles, or inverted triangles, respectively. Data expressed as mean ± SEM.

Both D0 Nec-1- and D3 Nec-1 treated islets on day 7 had a twofold increase in islet insulin content compared to untreated islets on day 3 (Figure 2). However, only the insulin content of D3 Nec-1 treated islets was significantly higher than the insulin content of untreated islets on day 7 (Figure 5).

**Figure 6:**
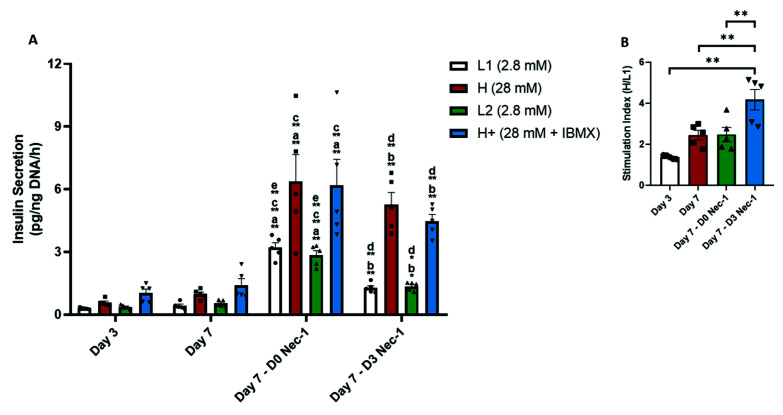
Function of PPIs in response to glucose challenge on day 3 and 7 of tissue culture in control media or media supplemented Nec-1 either immediately after islet isolation (D0 Nec-1) or on day 3 of tissue culture (D3 Nec-1). Islet function was evaluated using a glucose stimulated insulin release assay. A triplicate of 100 IEQs was incubated for 1 h in media with the corresponding order of glucose concentration: 2.8 mM (L1), 28 mM (H) 2.8 mM (L2), and 28 mM + 0.1mM IBMX (H+), glucose media. The concentration of secreted insulin from each media condition was quantified by ELISA and normalized to the sample DNA content. (**A**) Insulin secretion per ng DNA after incubation in varying concentrations of glucose media released by PPIs on day 3 and 7 of tissue culture. Individual data points for islet insulin secretion in 2.8 mM (L1), 28 mM (H) 2.8 mM (L2), and 28 mM + 0.1mM IBMX (H+), glucose media are shown within each bar graph as either circles, squares, triangles, or inverted triangles, respectively. (**B**) Stimulation index, calculated as the insulin secretion in H media over L1 media, of PPIs on day 3 and 7 of tissue culture. *n* = 5 for each group. * *p* < 0.05. ** *p* < 0.01. Individual data points for islets cultured in control media on day 3 (Day 3) or day 7 of tissue culture (Day 7) or in media supplemented with Nec-1 either immediately after islet isolation (D0 Nec-1) or on day 3 of tissue culture (D3 Nec-1) are shown within each bar graph as either circles, squares, triangles, or inverted triangles, respectively. Data expressed as mean ± SEM. a. Day 3 vs. Day 7-D0 Nec-1. b. Day 3 vs. Day 7-D3 Nec-1. c. Day 7 vs. Day 7-D0 Nec-1. d. Day 7 vs. Day 7-D3 Nec-1. e. Day 7-D0 Nec-1 vs. Day 7-D3 Nec-1.

In untreated islets, prolonged culture to 7 days did not improve the insulin secretion compared to day 3 of tissue culture in all glucose media conditions (Figure 6A). Islets cultured in media treated with Nec-1 either immediately after islet isolation or on day 3 of tissue culture had a 10.5- and 4-times higher insulin secretion in L1 glucose media than untreated islets on day 3, respectively (Figure 6A). The amount of insulin secreted in H glucose media by D0 Nec-1- and D3 Nec-1 treated islets was elevenfold and ninefold higher than untreated islets on day 3 (Figure 6A). Moreover, D0 Nec-1- and D3 Nec-1 treated islets had a 7.5- and 3.5-times higher amount of insulin secreted in L2 glucose media compared to untreated islets on day 3, respectively (Figure 6A). The insulin secretion in H+ glucose media from D0 Nec-1- and D3 Nec-1 treated was sixfold and fourfold higher than untreated islets on day 3, respectively (Figure 6A).

On day 7, D0 Nec-1- and D3 Nec-1 treated islets secreted sevenfold and threefold more insulin in L1 glucose media than untreated islets (Figure 6A). D0 Nec-1- and D3 Nec-1 treated islets had a sixfold and fivefold increase in the insulin secretion in H glucose media compared to untreated islets on day 7 (Figure 6A). The amount of insulin secreted in L2 glucose media from D0 Nec-1- and D3 Nec-1 treated islets was 5 and 2.5 times higher than islets cultured in untreated media, respectively (Figure 6A). Islets cultured in D0 Nec-1- or D3 Nec-1 treated media also had a fourfold and threefold increase in the amount of insulin secreted compared to untreated islets on day 7, respectively (Figure 6A).

In Nec-1 treated groups, islets treated immediately after islet isolation had a significantly higher amount of insulin secreted in L1 and L2 glucose media on day 7 compared to islets treated on day 3 (Figure 6A). Upon glucose challenge with H and H+ glucose media, there was no significant difference in the insulin secretion between D0 Nec-1- and D3 Nec-1 treated islets on day 7 (Figure 6A). Nec-1 supplementation on day 3 of tissue culture also significantly improved the stimulation index on day 7 in comparison to untreated islets on day 3, untreated islets on day 7, and D0 Nec-1 treated islets on day 7 (Figure 6B).

### 2.6. Nec-1 Supplementation on Day 3 of Tissue Culture Improves Islet Function after Transplantation into STZ-Induced Diabetic Mice

**Figure 7:**
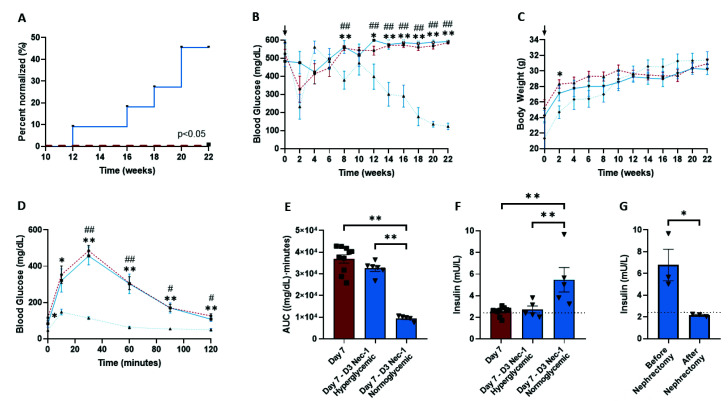
Long-term metabolic follow-up of diabetic athymic nude mice after islet transplantation with PPIs cultured for 7 days in control media (*n* = 10, dash red line) or media supplemented with Nec-1 on day 3 of tissue culture (hyperglycemic mice: *n* = 6, solid blue line; normoglycemic mice: *n* = 5, dotted blue line). Athymic nude mice were rendered diabetic by an intraperitoneal streptozotocin injection, transplanted with 5000 IEQs of PPIs under the kidney capsule, and followed for 22 weeks. (**A**) Percentage of mice achieving normoglycemia (blood glucose < 200 mg/dL for 2 consecutive weeks) after islet transplantation from week 10 to 22. (**B**) Average weekly non-fasting blood glucose measurements from 0 to 22 weeks after islet transplantation. (**C**) Average weekly body weight measurements from 0 to 22 weeks after islet transplantation. (**D**) Blood glucose measurements during an OGTT (3 mg/kg) at 22 weeks after islet transplantation. (**E**) Glucose clearance after an OGTT (3 mg/kg) at 22 weeks post-transplantation expressed as AUC. (**F**) Porcine insulin measurements in serum of mice at 22 weeks after islet transplantation. (**G**) Porcine insulin measurements in serum of normalized mice (*n* = 3) before and after nephrectomy of the graft-bearing kidneys at 22 weeks post-transplantation with islets cultured in media supplemented with Nec-1 on day 3 of tissue culture for 7 days. Downward arrows indicate time of implantation of an insulin pellet. Dotted black lines indicate the lower limit of the assay range (2.3 mU/L). * *p* < 0.05 (Day 7 vs. Day 7-D3 Nec-1-Normoglycemic). ** *p* < 0.01 (Day 7 vs. Day 7-D3 Nec-1-Normoglycemic). # *p* < 0.05 (Day 7-D3 Nec-1-Hyperglycemic vs. Day 7-D3 Nec-1-Normoglycemic). ## *p* < 0.01 (Day 7-D3 Nec-1-Hyperglycemic vs. Day 7-D3 Nec-1-Normoglycemic). Data expressed as mean ± SEM. Individual data points for islets cultured in control media (Day 7) or media supplemented with Nec-1 on day 3 of tissue culture (D3 Nec-1) are shown within each bar graph as either squares, inverted triangles, respectively.

The blood glucose levels of STZ-induced diabetic nude mice transplanted with D3 Nec-1 treated islets became normoglycemic as early as week 12, and these mice achieved normoglycemia significantly earlier and at a higher proportion than untreated islet recipients (Figure 7A). After 22 weeks, 45.5% of mice transplanted with D3 Nec-1 islets reached normoglycemia in comparison to 0% in mice that received untreated islets (Figure 7A). D3 Nec-1 treated islet recipients that achieved normoglycemia had significantly lower blood glucose levels at weeks 8 and 12 to 22 compared to hyperglycemic D3 Nec-1 treated islet recipients (Figure 7B). Similarly, the blood glucose levels of normoglycemic D3 Nec-1 treated islet recipients were significantly lower than untreated islet recipients at weeks 8 and 12 to 22 (Figure 7B). Furthermore, D3 Nec-1 treated islet recipients had, on average, significantly lower blood glucose levels at weeks 14 to 22 compared to untreated islet recipients (Supplementary Table S1).

During an OGTT, the blood glucose levels of mice transplanted with D3 Nec-1 treated islets that became normoglycemic were significantly lower than hyperglycemic mice after 30 min (Figure 7D). In addition, normoglycemic D3 Nec-1 treated islet recipients had significantly lower blood glucose levels at all timepoints compared to untreated islet recipients during an OGTT (Figure 7D). An oral glucose load was cleared nearly 4 and 3.5 times faster by normoglycemic D3 Nec-1 treated islet recipient mice than untreated islet- and hyperglycemic D3 Nec-1 treated islet recipient mice, respectively (Figure 7E). On average, mice transplanted with D3 Nec-1 treated islets had significantly lower blood glucose levels at all timepoints throughout an OGTT and were 1.7 times more glucose tolerant than mice transplanted with untreated islets (Supplementary Figure S4F–I).

**Figure 8:**
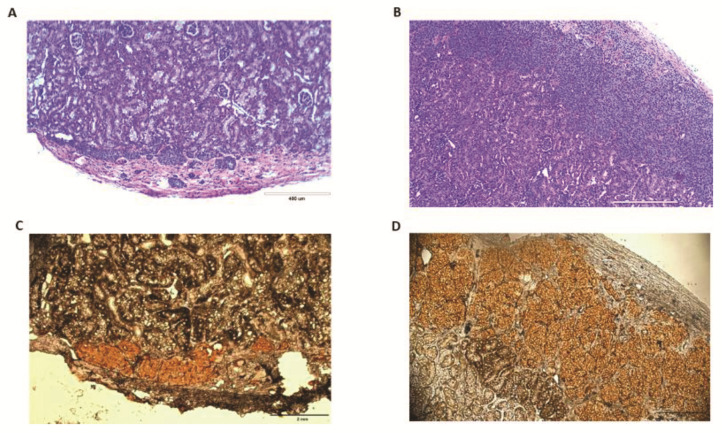
Histology and immunohistochemistry of graft-bearing kidneys at 22 weeks after transplantation with PPIs cultured for 7 days in control media or media supplemented with Nec-1 on day 3 of tissue culture. Graft-bearing kidneys were recovered at 22 weeks after islet transplantation for histological and immunohistochemical analysis. (**A**) Representative photomicrograph of graft-bearing kidneys stained with hematoxylin and eosin from hyperglycemic mice transplanted with PPIs cultured for 7 days in control media. (**B**) Representative photomicrograph of graft-bearing kidneys stained with hematoxylin and eosin from normoglycemic mice transplanted with PPIs cultured for 7 days in media supplemented with Nec-1 on day 3 of tissue culture. (**C**) Representative photomicrograph of graft-bearing kidneys stained with insulin from hyperglycemic mice transplanted with PPIs cultured for 7 days in control media. (**D**) Representative photomicrograph of graft-bearing kidneys stained with insulin from normoglycemic mice transplanted with PPIs cultured for 7 days in media supplemented with Nec-1 on day 3 of tissue culture. Scale bar = 400 μm (Figure 8A,B) and 2 mm (Figure 8C,D).

In addition, the level of plasma porcine insulin from D3 Nec-1 treated islet recipients was approximately 1.5 times higher than untreated islet recipients (Supplementary Figure S4E). Normoglycemic mice transplanted with D3 Nec-1 treated islets had a twofold increase in the level of plasma porcine insulin compared to hyperglycemic mice transplanted with either untreated- or D3 Nec-1 treated islets (Figure 7F). A return to hyperglycemia was observed in all normoglycemic D3 Nec-1 treated islet recipients after the nephrectomy of the graft-bearing kidney, indicating that the attainment of normoglycemia was due to the porcine islet graft and not to beta-cell regeneration (Supplementary Figure S4C). After survival nephrectomy of the graft-bearing kidney from normoglycemic D3 Nec-1 treated islet recipients, the level of plasma porcine insulin fell below the lower limit of the ELISA range and were significantly lower than the plasma porcine insulin level before the nephrectomy (Figure 7G). H&E staining and insulin immunostaining of the graft-bearing kidneys removed from mice transplanted with either untreated islets or D3 Nec-1 treated islets confirmed the presence of islets and insulin-positive cells underneath the kidney capsule (Figure 8A–D).

## 3. Discussion

Previously, we have shown that Nec-1 improved islet development and function in vitro when added to culture media immediately after islet isolation [15]. In the current study, the effects that Nec-1 supplementation on day 3 of tissue culture have on PPIs in vitro and in vivo after transplantation in STZ-induced diabetic mice were examined. Our results clearly showed that Nec-1 supplementation on day 3 of culture significantly improved islet recovery, insulin content, endocrine cellular composition, GLUT2 expression, differentiation and proliferation of endocrine cells, insulin secretion capacity, and transplantation outcomes compared to untreated islets. Diabetic mice receiving islets treated with Nec-1 on day 3 of tissue culture not only reached normoglycemia sooner and exhibited lower blood glucose levels, but also had better glucose clearance and higher circulating plasma porcine insulin than mice receiving untreated islets. Moreover, Nec-1 added on day 3 of tissue culture was superior to that added immediately after islet isolation as evidenced by a significant improvement in islet recovery, proportion of delta cells, differentiation and proliferation of beta cells, and stimulation index during glucose challenge after 7 days of tissue culture.

Surprisingly, the loss of islets during culture was ameliorated after the delayed supplementation of Nec-1 on day 3 of tissue culture but not immediately after islet isolation. While the majority of studies started Nec-1 treatment on the same day of an experiment, our novel findings support evidence from a previous study that delaying Nec-1 treatment for up to 72 h improved the survival rate of mice exposed to total body irradiation [19]. Another study has shown that Nec-1 supplementation started 5 days after the formation of Aβ aggregates in brain cells from monomeric Aβ42 peptides separated pre-formed Aβ aggregates and reduced the buildup of Aβ fibrils [20]. Our current data and previous works suggest that the timing of Nec-1 supplementation to tissue culture can notably affect the effectiveness of Nec-1.

Even though Nec-1 supplementation has been reported to increase islet cell viability, our present study did not detect any significant difference in the viability of islets using three different assays [13]. A recent study demonstrated that the levels of dead cells assessed by Sytox staining in βTC-6 beta cells, INS-1/832 beta cells, and isolated murine islet cells were unaffected by Nec-1 supplementation after 24-h incubation in standard cell culture conditions [13]. However, Nec-1 supplementation blocked cell death when islets were cultured under low oxygen condition or with nitric oxide donors [13,14]. Our data support these observations as islets in the current study were cultured in an optimal media and condition, while other studies exposed Nec-1 treated islet cells to considerable stress.

Nec-1 supplementation on day 3 of tissue culture, but not immediately after islet isolation, also significantly improved the islet insulin content compared to untreated islets on day 7 of tissue culture. This increase in the islet insulin content is most likely due to the marked development of beta cells in Nec-1 treated islets [21]. A plausible explanation behind the effects of Nec-1 supplementation to increase the beta-cell content is that islets are afflicted with substantial damage from islet isolation and culture, resulting in the upregulation of pro-inflammatory mediators [22,23]. Islet inflammation not only increases islet cell death but also causes the beta-cell dedifferentiation [24,25]. As Nec-1 has been reported to block DAMP-induced inflammatory responses, Nec-1 supplementation during islet culture may support the differentiation of beta cells by inhibiting inflammation [13,14]. Another possible reason for this increase in endocrine cellular content is that the generation of new pancreatic endocrine cells involves both progenitor cell differentiation and endocrine cell proliferation [26,27]. As the transcription factor Ngn3 has been reported to be suppressed after pancreatic progenitor cells differentiate into mature endocrine cells, the decrease in the level of Ngn3-positive pancreatic progenitor cells with culture time in our study is consistent with the increase in the proportion of major endocrine cells [28,29]. This finding is also supported by a previous study, showing a downregulation of Ngn3 expression in human embryonic stem cells (hESCs) as the expression of insulin, glucagon, and somatostatin increases after 24 days of differentiation culture [30]. The increase in Nkx6.1 expression over a 16-day differentiation culture of hESCs has been correlated with the expansion of beta cells [30]. This finding is in line with our results that the levels of both Nkx6.1-positive cells and insulin-positive beta cells were increased in islets treated with Nec-1 on day 3 of tissue culture. Similar to our current data that the higher level of proliferating Ki67-positive beta cells was partially responsible for the increased proportion of beta cells in islets treated with Nec-1 on day 3 of tissue culture, others have reported that the enhanced proliferative capacity of islet beta cells augments the expansion of beta-cell mass and islet insulin secretion capacity [31]. Moreover, delaying the supplementation of Nec-1 to day 3 of tissue culture significantly improved beta-cell proliferation compared to day 0. As Nec-1 has been shown to be ineffective and even have toxic effects when its use is not optimized, a possible reason for this finding is that the prolonged culture of islets in media supplemented with Nec-1 immediately after isolation for 7 days could be damaging to islet cells, especially beta cells, which have been determined to be more vulnerable to insults than other types of islet cells [32,33,34,35]. This notion is further supported by previous studies, demonstrating that Nec-1 was primarily utilized for only 24 h and up to 5 days at most for in vitro culture [10,20,36]. In accordance with previous findings that the proliferation of mature delta cells is responsible for the formation of new delta cells, islets treated with Nec-1 on day 3 of tissue culture had a significant growth in both proliferating delta cells and delta-cell mass compared to untreated islets [37]. Future studies examining how the inhibitory action of Nec-1 on inflammation contributes to the differentiation, proliferation, and expansion of pancreatic islet endocrine cells will assist to elucidate the underlying molecular mechanism.

As GLUT2 is critical for the sensing and uptake of glucose in the beta cells of porcine pancreatic islets, the significantly lower insulin secretion capacity of young porcine islets is most likely due to the twofold decrease in GLUT2 expression compared to adult porcine islets [38]. The enhancement in the expression of GLUT2 in beta cells combined with a higher proportion of endocrine cells and insulin content most likely accounts for the superior glucose-stimulated insulin secretion capacity of Nec-1 treated islets compared to untreated islets. These results are in line with previous studies, demonstrating that the increase in GLUT2 expression, insulin content, and composition of endocrine cells result in significantly higher insulin output [21,39]. Furthermore, the amounts of insulin secreted by islets treated with Nec-1 either immediately after islet isolation or on day 3 of tissue culture were comparable to adult porcine islets in both basal and stimulated glucose conditions [38]. This improvement in the islet insulin secretion may be due to the effects of Nec-1 to suppress the cytoprotective factor prostaglandin E2 (PGE2), which acts as an inhibitor of glucose-stimulated insulin secretion in isolated pancreatic islets [40,41]. In addition to the improvement in insulin secretion, the twofold increase in the stimulation index of islets treated with Nec-1 on day 3 of tissue culture indicated a higher responsiveness to glucose stimulation and prompted us to evaluate the function of these islets in STZ-induced diabetic mice.

Our findings that Nec-1 supplementation on day 3 of tissue culture significantly enhanced the function of PPIs after transplantation are concordant with previous reports, where the enhanced in vitro maturation of young porcine islets leads to improved functional outcomes [21]. While the majority of these studies required islets to be cultured for a prolonged period of up to 27 days, the delayed supplementation of Nec-1 alone for only 3 days augmented the glycemic control and glucose clearance of islet recipients [8]. Moreover, this is the first study, to our knowledge, to show the lasting post-transplantation effects of using a cytoprotective agent during in vitro maturation culture on significantly improving the circulating plasma porcine insulin secreted by islets during the non-fasting state. The time to achieve normoglycemia and proportion of normalized mice in our study is also comparable to previous studies that transplanted young porcine islets into chemical-induced diabetic mice. For example, Hayward et al. showed that 50% of mice co-transplanted with islets and mesenchymal stem cells were normalized at 19.5 weeks while none of the mice transplanted with islets alone reached normoglycemia [42]. In another study by Hassouna et al., neonatal porcine islets cultured for 20 days in a standard islet culture media could only restore normoglycemia in half of the islet recipients after 25 weeks [21]. Similarly, Jimenez-Vera et al. have observed that the normalization rate was merely 35% at 100 days in mice transplanted with islets cultured for 6 days [8]. In addition to the inadequate amount of insulin-producing beta cells, another reason for the delay in time to normalization is that diabetic rodents are resistant to porcine insulin and may require up to 40 times the dose used in humans [43]. Since prolonged culture of young porcine islets in standard islet culture media for more than 7 days has been reported to improve the time to achieve normoglycemia in streptozotocin-induced diabetic mice, determining whether the time in culture could impact the in vivo function of Nec-1 treated PPIs will further optimize islet maturation and improve functional outcomes after transplantation [8].

In summary, the current study presents the novel effects of Nec-1 supplementation on day 3 of tissue culture to enhance both the in vitro and in vivo function of PPIs after 7 days of tissue culture. Future studies will focus on further elucidating the molecular mechanism by which Nec-1 improves islet development and function as well as examining the efficacy of Nec-1 treated PPIs after transplantation into large animal models of Type 1 diabetes. Since young porcine islets represent an unlimited donor source, Nec-1 supplementation on day 3 of tissue culture will augment islet quantity and quality prior to transplantation.

## 4. Research Design and Methods

### 4.1. Animals

The Institutional Animal Care and Use Committee at the University of California, Irvine approved all described animal procedures. Pre-weaned Yorkshire piglets (S&S Farms) aged 8–15 days old were used as islet donors. Male athymic nude mice (Charles River) at 8 weeks old served as xenotransplant recipients.

### 4.2. PPI Isolation

PPIs were isolated from pancreata of 8–15-day-old, pre-weaned Yorkshire piglets as previously described [44]. Briefly, pancreata were procured in less than 10 min and stored into cold HBSS (cold ischemic time < 1 h). Pancreata were minced into 1 mm^3^ pieces and digested with Sigma Type V Colla-genase (cat#C8051, Sigma Aldrich, Buchs, Switzerland) dissolved in HBSS (2.5 mg/mL) in 37 °C, 100 rpm shaking water bath for 15 min. After quenching with HBSS supplemented with 1% porcine serum (cat#26250084, Gibco, Waltham, MA, USA), digested tissues were filtered through a 500 μm metal mesh.

### 4.3. Islet Tissue Culture and Nec-1 Supplementation

After islet isolation, PPIs were cultured in islet maturation media (IMM) for 7 days [45]. Untreated islets cultured in IMM alone served as a control (*n* = 5), whereas Nec-1 treated islets were cultured in IMM supplemented with Nec-1 (100 µM; cat#ab141053, Abcam, Cambridge, UK) either immediately after islet isolation (D0 Nec-1, *n* = 5) or on day 3 (D3 Nec-1, *n* = 5) [16]. A full media change was performed on day 1, and a half media change was done every 48 h thereafter.

### 4.4. Islet Recovery and Viability

A 100 uL aliquot of islets was collected for staining with 1 mL dithizone (cat#150999, MP Biomedicals, Santa Ana, CA, USA) for 5 min and counted on a standard stereo microscope with a 10× eyepiece graticule to determine the islet equivalence (IEQ) [44]. The islet recovery on day 7 was expressed as the percentage of IEQ per gram of pancreatic tissue normalized to day 3 of control islets.

### 4.5. Flow Cytometric Analysis of Islets

Flow cytometry was used to quantify islet cellular viability, cellular composition, GLUT2 expression, progenitor cell differentiation, and endocrine cell proliferation [46]. In brief, samples of single cell suspension were obtained by dissociating 3000 IEQs in Accutase (cat#AT104, Innovative Cell Technologies, Lucerne, Switzerland) and incubated in 7-aminoactinomycin D dye on ice (7-AAD, 10 μg/mL; cat#A1310, Invitrogen, Basel, Switzerland) for 30 min. Cell samples were then fixed with 4% paraformaldehyde for 10 min and permeabilized using Intracellular Staining Permeabilization Wash Buffer (cat#421002, BioLegend, San Diego, CA, USA) for 15 min on ice. After incubation in Protein Block (cat#ab64226, Abcam, Cambridge, UK) for 30 min on ice, cell samples were stained with fluorescently conjugated antibodies diluted in Intracellular Staining Permeabilization Wash Buffer supplemented with 0.5% bovine serum albumin (cat#BAL62-0500, Equitech-Bio, Kerrville, TX, USA) for 30 min on ice. All antibodies are listed in Supplementary Table S2 Stained cell samples were analyzed on a flow cytometer (NovoCyte 3000VYB, ACEA Biosciences, San Diego, CA, USA) and quantified using FlowJo software (FlowJo, BD Life Sciences, Franklin Lakes, NJ, USA). Gating controls included unstained, single-stained, fluorescence minus one, and matching isotype control samples.

### 4.6. In Vitro Islet Function

Glucose-stimulated insulin release assay was used to evaluate islet function [44]. A triplicate of 100 IEQs per sample was incubated in the following order of glucose media for 1 h each: low glucose (2.8 mM; L1), high glucose (28 mM; H), low glucose (2.8 mM; L2) and in high glucose plus 3-isobutyl-1-methylxanthine9 (28 mM + 0.1 mM IBMX; H+). The insulin concentration was determined using a porcine insulin ELISA kit (cat#10-1200-01, Mercodia, Uppsala, Sweden) and normalized to the DNA content quantified by a fluorescent dsDNA stain kit (cat#Q32850, Molecular Probes, Eugene, OR, USA) [47]. The stimulation index was calculated as the in-sulin secretion in H glucose media over L1 glucose media.

### 4.7. Islet Insulin Content

150 IEQs were lysed (10 mM Tris-HCl, 1mM EDTA, 1% Triton-X100, pH 8) and sonicated (Sonics Vi-braCell Ultrasonic Processor Model VC70T, Sonics Materials, Newtown, CT, USA) for 30 s on ice. After centrifugation at 1400× *g* for 15 min at 4 °C, the insulin content from the supernatant was determined using a porcine insulin ELISA kit and normalized to the DNA content [47].

### 4.8. Islet Transplantation and Metabolic Follow-Up

One week before islet transplantation, mice were rendered diabetic via a single intraperitoneal injection of streptozotocin (STZ, 200 mg/kg; cat#S0130-100MG, Sigma Aldrich; Buchs, Switzerland). Diabetic mice were confirmed by two consecutive measurements of non-fasting blood glucose levels ≥ 350 mg/dL from the tail vein (CON-TOUR^®^NEXT glucometer, Ascensia Diabetes Care, Basel, Switzerland). On day 7, 5000 IEQs of control or D3 Nec-1 treated PPIs were transplanted under the kidney capsule of diabetic mice [48]. Due to the delayed in vivo function of PPIs, each mouse was implanted subcutaneously with an insulin pellet (cat#Pr-1-B, LinShin, Toronto, ON, Canada) that lasted approximately 4 weeks. All mice were monitored for non-fasting blood glucose levels and bodyweight on a weekly basis for 22 weeks. Normoglycemia was defined as maintaining blood glucose levels of ≤200 mg/dL for two consecutive weeks. After 22 weeks, all mice underwent an oral glucose tolerance test (OGTT, 3 mg/kg) [48]. Following OGTT, a survival nephrectomy of the graft-bearing kidney was performed on normoglycemic mice to confirm the return of hyperglycemia (non-fasting blood glucose level ≥ 350 mg/dL). Blood samples from the retro orbital plexus were obtained to measure circulating plasma porcine insulin levels before survival nephrectomy or euthanasia. After the return of hyperglycemia, blood samples were collected again to confirm the absence of plasma porcine insulin levels. Following centrifugation and separation from whole blood samples, the porcine insulin concentration in plasma samples was analyzed using a porcine insulin ELISA kit.

### 4.9. Histological and Immunohistochemical Analysis

The graft-bearing kidneys were used for histological and immunohistochemical analysis to confirm the presence of porcine islets and insulin-positive beta cells underneath the kidney capsule. After fixation in 10% neutral buffered formalin for 48 h, tissues underwent serial dehydration and were embedded with paraffin. Tissues were then cut into 5 µm tissue sections and stained with hematoxylin and eosin (H&E) using standard techniques.

An immunohistochemistry HRP kit (cat#K0675, DAKO, Glostrup, Denmark) was used to detect insulin-positive beta cells according to the manufacturer’s protocol [49]. In brief, 5 µm tissue sections were deparaffinized and rehydrated. Antigen retrieval was induced by heat in a citrate buffer at pH 6.0 for 15 min. Tissues sections were then incubated in protein block (cat#HK112-9K, BioGenex, San Ramon, CA, USA) for 1 h and 3% hydrogen peroxide for 10 min at room temperature. Following blocking, tissue sections were incubated overnight at 4 °C with insulin primary antibody (1:2000; cat#3014S, Cell Signaling Technology, Danvers, MA, USA) [50]. After washing, tissue sections were incubated in ready-to-use secondary antibody solution for 15 min and DAB peroxidase substrate-chromogen solution for 3–5 min at room temperature. Tissue sections were then counterstained with Meyer’s hematoxylin and sealed with coverslips. All slides were imaged on an inverted microscope (Nikon Ti-E Widefield microscope, Nikon Instruments, Tokyo, Japan). Cells with a cytoplasmic brown staining were identified as insulin-positive beta cells.

### 4.10. Statistical Analysis

All data are presented as mean ± the standard error of mean (SEM). Statistical significance was evaluated using a one-way ANOVA followed by a post-hoc Tukey’s HSD test. A log-rank (Mantel-Cox) test was used to determine the statistical significance in the percentage of mice achieving normoglycemia. A *p* < 0.05 was considered to be statistically significant. All data analysis was conducted on Prism 8.0.1 (For Software, version 8.4, San Diego, CA, USA).

## Supplementary Materials

The following are available online at https://www.mdpi.com/article/10.3390/ijms22168367/s1.
