# Supplementary material for: Necrostatin-1 Supplementation to Islet Tissue Culture Enhances the In-Vitro Development and Graft Function of Young Porcine Islets
Source: Int J Mol Sci. 2021 Aug 4;22(16):8367. doi: 10.3390/ijms22168367 (PMC8394857; doi:10.3390/ijms22168367)
Supplement: Supplementary file 1 [file ijms-22-08367-s001.zip › Nec-1 D3 vs. D0 - Online Supplement Materials - IJMS.pdf]

Supplementary Table S1. List of all antibodies used for the analysis of islets via flow cytometry.

Antibody	Catalog # and Manufacturer	Dilution	Target
PE-conjugated anti-insulin	Cat #8508, CST, Danvers, MA	1:50	Beta cells
APC-conjugated anti-glucagon	Cat #NBP2-21803AF647, Novus Biological	1:100	Alpha cells
Alexa Fluor 488-conjugated anti-somatostatin	Cat #566032, BD Biosciences	1:50	Delta cells
FITC-conjugated anti-GLUT2	Cat #FAB1414G-100UG, Novus Biological	1:100	Double staining with anti-insulin antibody to identify GLUT2-positive, insulin-positive beta cells
FITC-conjugated anti-Neurogenin 3 (Ngn3)	Cat #bs-0922R, Bioss	1:133	Ngn3-positive pancreatic progenitor cells
APC-conjugated anti-Nkx6.1	Cat #563338, BD Pharmingen	1:33	Nkx6.1-positive pancreatic progenitor cells
PE-conjugated anti-Ki-67	Cat #12-5698-82, Invitrogen	1:100	Double staining with anti-glucagon and anti-somatostatin antibodies to identify Ki-67-positive, glucagon-positive alpha cells and Ki-67-positive, somatostatin-positive delta cells
eFluor 660-conjugated anti-Ki-67	Cat #50-5698-82, Invitrogen	1:100	Double staining with anti-insulin antibody to identify Ki-67-positive, insulin-positive beta cells

Supplementary Table S2. Values of results mentioned in the manuscript

Figure S1
Untreated islets on day 3 of culture (control): 100%
Untreated islets on day 7 of culture: 50.7 ± 8.3% of control
D0 Nec-1 treated islets on day 7 of culture: 40.6 ± 10.6% of control
D3 Nec-1 treated islets on day 7 of culture: 88.7 ± 3.3% of control

Figure S2
Untreated islets on day 3 of culture: 31.3 ± 2.5 pg/ng DNA
Untreated islets on day 7 of culture: 40.3 ± 7.1 pg/ng DNA
D0 Nec-1 treated islets on day 7 of culture: 105.1 ± 25.2 pg/ng DNA
D3 Nec-1 treated islets on day 7 of culture: 116.5 ± 22.4 pg/ng DNA

Figure S3A-B
Untreated islets on day 3 of culture: 4.9 ± .3%
Untreated islets on day 7 of culture: 7.9 ± 1.1%
D0 Nec-1 treated islets on day 7 of culture: 15.1 ± 1.6%
D3 Nec-1 treated islets on day 7 of culture: 17.5 ± .6%

Figure S3C-D
Untreated islets on day 3 of culture: 21.4 ± 4.8%
Untreated islets on day 7 of culture: 31.4 ± 3.0%
D0 Nec-1 treated islets on day 7 of culture: 43.6 ± 8.3%
D3 Nec-1 treated islets on day 7 of culture: 46.2 ± 3.9%

Figure S3E-F
Untreated islets on day 3 of culture: 2.3 ± .6%
Untreated islets on day 7 of culture: 6.7 ± .6%
D0 Nec-1 treated islets on day 7 of culture: 15.4 ± 1.2%
D3 Nec-1 treated islets on day 7 of culture: 16.1 ± .8%

Figure S3G-H
Untreated islets on day 3 of culture: 1.10 ± .1%
Untreated islets on day 7 of culture: 2.2 ± .2%
D0 Nec-1 treated islets on day 7 of culture: 2.5 ± .2%
D3 Nec-1 treated islets on day 7 of culture: 3.0 ± .3%

Figure S4A-B
Untreated islets on day 3 of culture: 82.4 ± 6.3%

Untreated islets on day 7 of culture: 62.6 ± 6.1%
D0 Nec-1 treated islets on day 7 of culture: 43.2 ± 4.1%
D3 Nec-1 treated islets on day 7 of culture: 48.7 ± 3.0%

Figure S4C-D
Untreated islets on day 3 of culture: 6.0 ± 1.3%
Untreated islets on day 7 of culture: 14.3 ± 1.0%
D0 Nec-1 treated islets on day 7 of culture: 6.5 ± 1.2%
D3 Nec-1 treated islets on day 7 of culture: 16.4 ± 1.2%

Figure S5A-B
Untreated islets on day 3 of culture: 28.4 ± 2.5%
Untreated islets on day 7 of culture: 48.6 ± 4.7%
D0 Nec-1 treated islets on day 7 of culture: 53.4 ± 9.2%
D3 Nec-1 treated islets on day 7 of culture: 87.9 ± 2.0%

Figure S5C-D
Untreated islets on day 3 of culture: 68.4 ± 4.0%
Untreated islets on day 7 of culture: 68.8 ± 1.8%
D0 Nec-1 treated islets on day 7 of culture: 85.6 ± 2.7%
D3 Nec-1 treated islets on day 7 of culture: 92.0 ± 3.9%

Figure S5E-F
Untreated islets on day 3 of culture: 55.5 ± 9.6%
Untreated islets on day 7 of culture: 40.0 ± 4.4%
D0 Nec-1 treated islets on day 7 of culture: 69.7 ± 8.8%
D3 Nec-1 treated islets on day 7 of culture: 75.5 ± 8.9%

Figure S6A
Untreated islets on day 3 of culture: L1: .3 ± .02 pg/ng DNA/h, H: .6 ± .07 pg/ng DNA/h, L2: .4 ± .04 pg/ng DNA/h, and H+: 1.0 ± .2 pg/ng DNA/h
Untreated islets on day 7 of culture: L1: .4 ± .07 pg/ng DNA/h, H: 1.0 ± .1 pg/ng DNA/h, L2: .5 ± .06 pg/ng DNA/h, and H+: 1.4 ± .3 pg/ng DNA/h
D0 Nec-1 treated islets on day 7 of culture: L1: 3.2 ± .2 pg/ng DNA/h, H: 6.4 ± 1.3 pg/ng DNA/h, L2: 2.9 ± .2 pg/ng DNA/h, and H+: 6.2 ± 1.3 pg/ng DNA/h
D3 Nec-1 treated islets on day 7 of culture: L1: 1.3 ± .1 pg/ng DNA/h, H: 5.3 ± .6 pg/ng DNA/h, L2: 1.4 ± .09 pg/ng DNA/h, and H+: 4.5 ± .3 pg/ng DNA/h

Figure S6B
Untreated islets on day 3 of culture: 1.4 ± .04

Untreated islets on day 7 of culture: 2.5 ± .2
D0 Nec-1 treated islets on day 7 of culture: 2.5 ± .3
D3 Nec-1 treated islets on day 7 of culture: 4.2 ± .5

Figure S7A
Untreated islet recipient mice: Week 12: .0%, Week 16: .0%, Week 18: .0%, Week 20: .0%, Week 22: .0%
D3 Nec-1 treated islet recipient mice: Week 12: 9.1%, Week 16: 18.2%, Week 18: 27.3%, Week 20: 45.5%, Week 22: 45.5%

Figure S7E
Untreated islet recipient mice: 36862.0 ± 1908.0 (mg/dL)·minutes
Hyperglycemic D3 Nec-1 treated islet recipient mice: 32472.0 ± 1307.0 (mg/dL)·minutes
Normoglycemic D3 Nec-1 treated islet recipient mice: 9517.0 ± 514.4 (mg/dL)·minutes

Figure S7F
Untreated islet recipient mice: 2.6 ± .1 mU/L
Hyperglycemic D3 Nec-1 treated islet recipient mice: 2.7 ± .3 mU/L
Normoglycemic D3 Nec-1 treated islet recipient mice: 5.5 ± 1.1 mU/L

Figure S7G
Before survival nephrectomy: 6.8 ± 1.5 mU/L
After survival nephrectomy: 2.2 ± .07 mU/L

Supplementary Figure S1
Untreated islets on day 3 of culture: 95.6 ± 1.2%
Untreated islets on day 7 of culture: 94.8 ± .9%
D0 Nec-1 treated islets on day 7 of culture: 97.0 ± .4%
D3 Nec-1 treated islets on day 7 of culture: 97.3 ± .3%

Supplementary Figure S2
Untreated islets on day 3 of culture: 91.3 ± 2.2%
Untreated islets on day 7 of culture: 88.8 ± 2.8%
D0 Nec-1 treated islets on day 7 of culture: 92.4 ± .6%
D3 Nec-1 treated islets on day 7 of culture: 90.8 ± .7%

Supplementary Figure S3
Untreated islets on day 3 of culture: 354.3 ± 22.3 nmol/min·mg DNA
Untreated islets on day 7 of culture: 338.8 ± 51.6 nmol/min·mg DNA
D0 Nec-1 treated islets on day 7 of culture: 361.2 ± 41.8 nmol/min·mg DNA

D3 Nec-1 treated islets on day 7 of culture: 310.0 ± 52.2 nmol/min·mg DNA

Supplementary Figure S4E
Untreated islet recipient mice: 2.6 ± .1 mU/L
D3 Nec-1 treated islet recipient mice: 4.1 ± .7 mU/L

Supplementary Figure S4I
Untreated islet recipient mice: 36862.0 ± 1908.0 (mg/dL)·minutes
D3 Nec-1 treated islet recipient mice: 22038.0 ± 3685.0 (mg/dL)·minutes

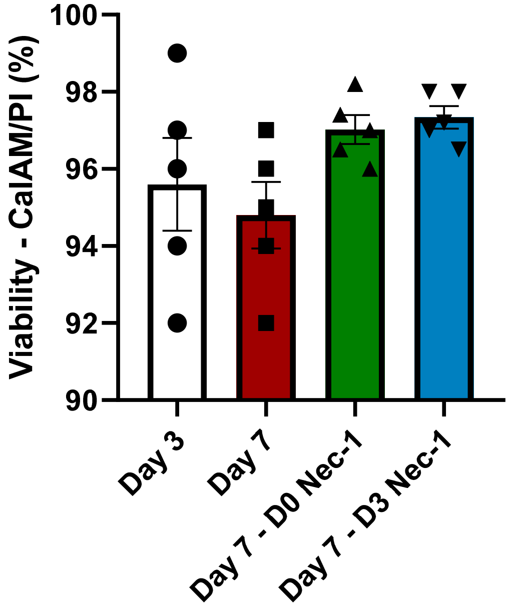

Supplementary Figure S1. Islet viability on day 3 and 7 of tissue culture in control media or media supplemented with Nec-1 either immediately after islet isolation (D0 Nec-1) or on day 3 of tissue culture (D3 Nec-1). 100 IEQs were stained with Calcein AM (CalAM) for live cells and propidium iodide (PI) for dead and dying cells for 30 minutes on day 3 and 7 of tissue culture. The islet viability was calculated by the equation: CalAM-positive cells/(CalAM-positive cells + PI-positive cells) x 100. n=5 for each group. Data expressed as mean ± SEM.

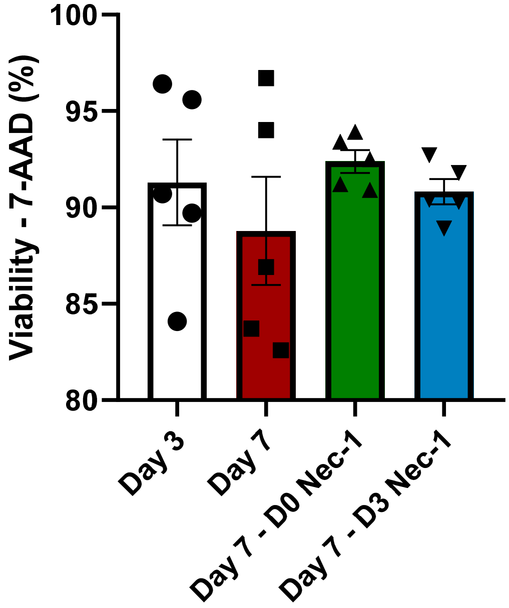

Supplementary Figure S2. Flow cytometric analysis of the viability of PPIs on day 3 and 7 of tissue culture in control media or media supplemented with Nec-1 either immediately after islet isolation (D0 Nec-1) or on day 3 of tissue culture (D3 Nec-1). Islets were dissociated on day 3 and 7 of tissue culture using Accutase, stained with 7-AAD viability dye, and analyzed by flow cytometry. n=5 for each group. Data expressed as mean ± SEM.

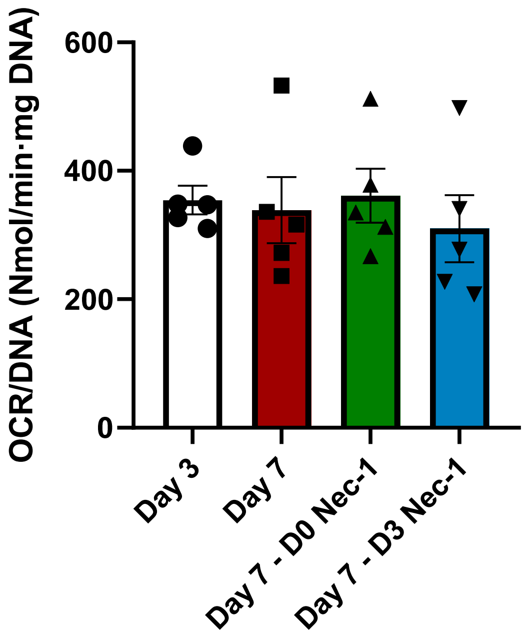

Supplementary Figure S3. Oxygen consumption rate of PPIs on day 3 and 7 of tissue culture in control media or media supplemented with Nec-1 either immediately after islet isolation (D0 Nec-1) or on day 3 of tissue culture (D3 Nec-1). 200 IEQs per isolation was evaluated for the oxygen consumption rate on day 3 and 7 of tissue culture using a fiber optic sensor monitoring system and expressed as oxygen consumption rate normalized to the total DNA. n=5 for each group. Data expressed as mean ± SEM.

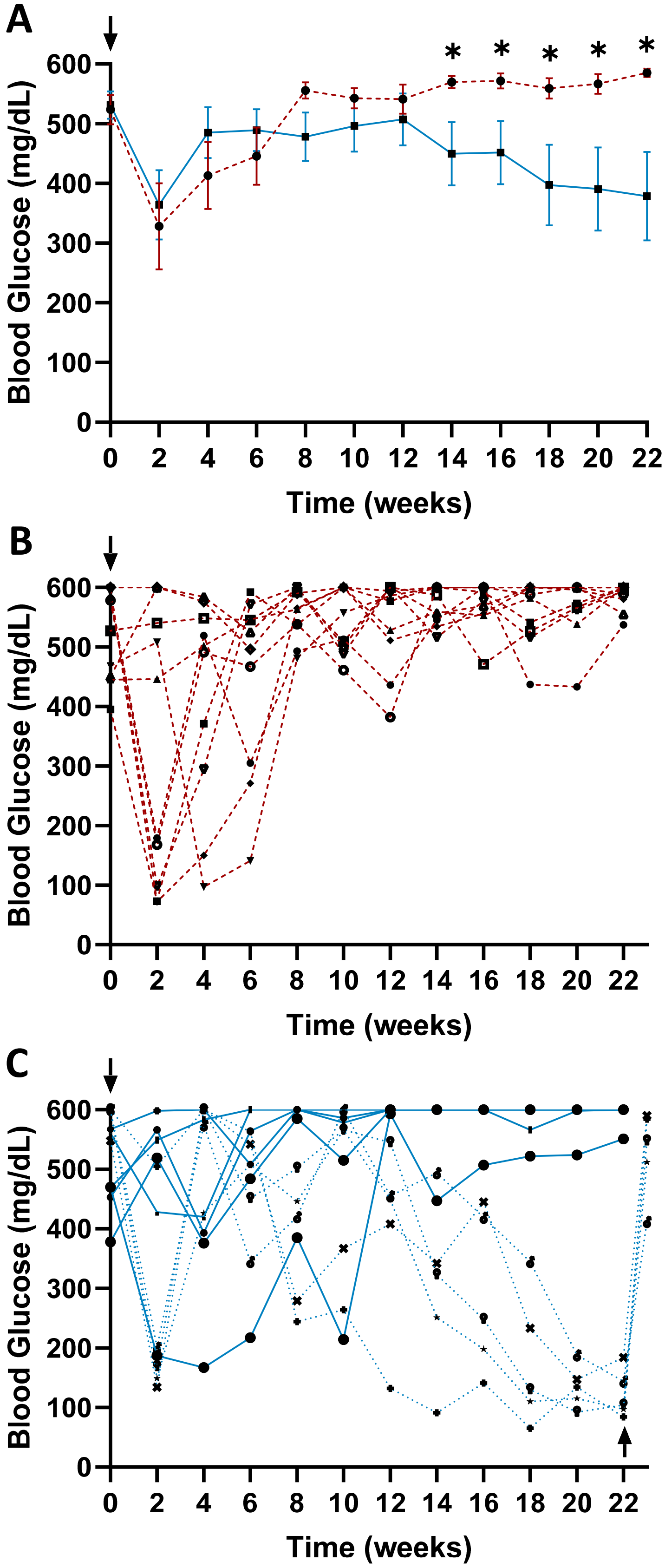
A
Blood Glucose (mg/dL)
Time (weeks)
B
Blood Glucose (mg/dL)
Time (weeks)
C
Blood Glucose (mg/dL)
Time (weeks)

D

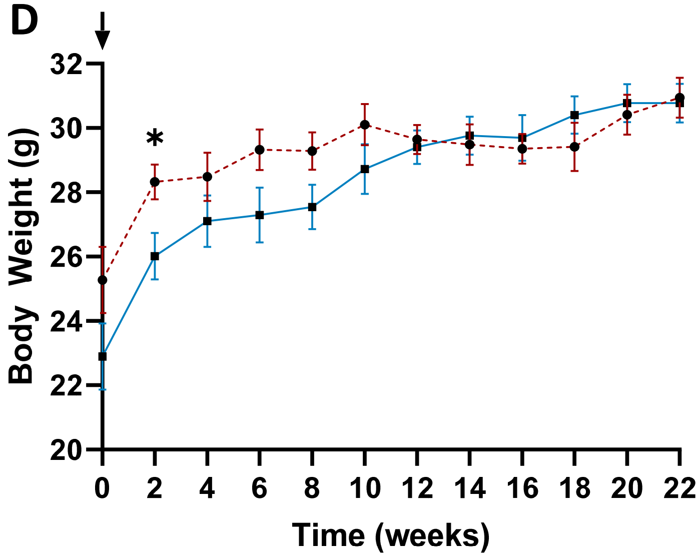

32
30
28
26
24
22
20
Body Weight (g)
*
0
2
4
6
8
10
12
14
16
18
20
22
Time (weeks)

E

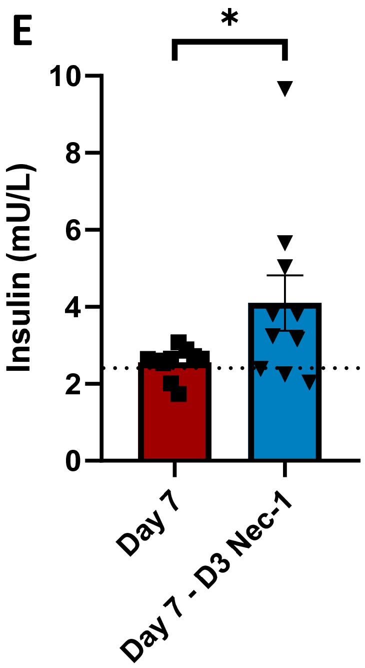

*
10
8
6
4
2
0
Insulin (mU/L)
Day 7
Day 7 - D3 Nec-1

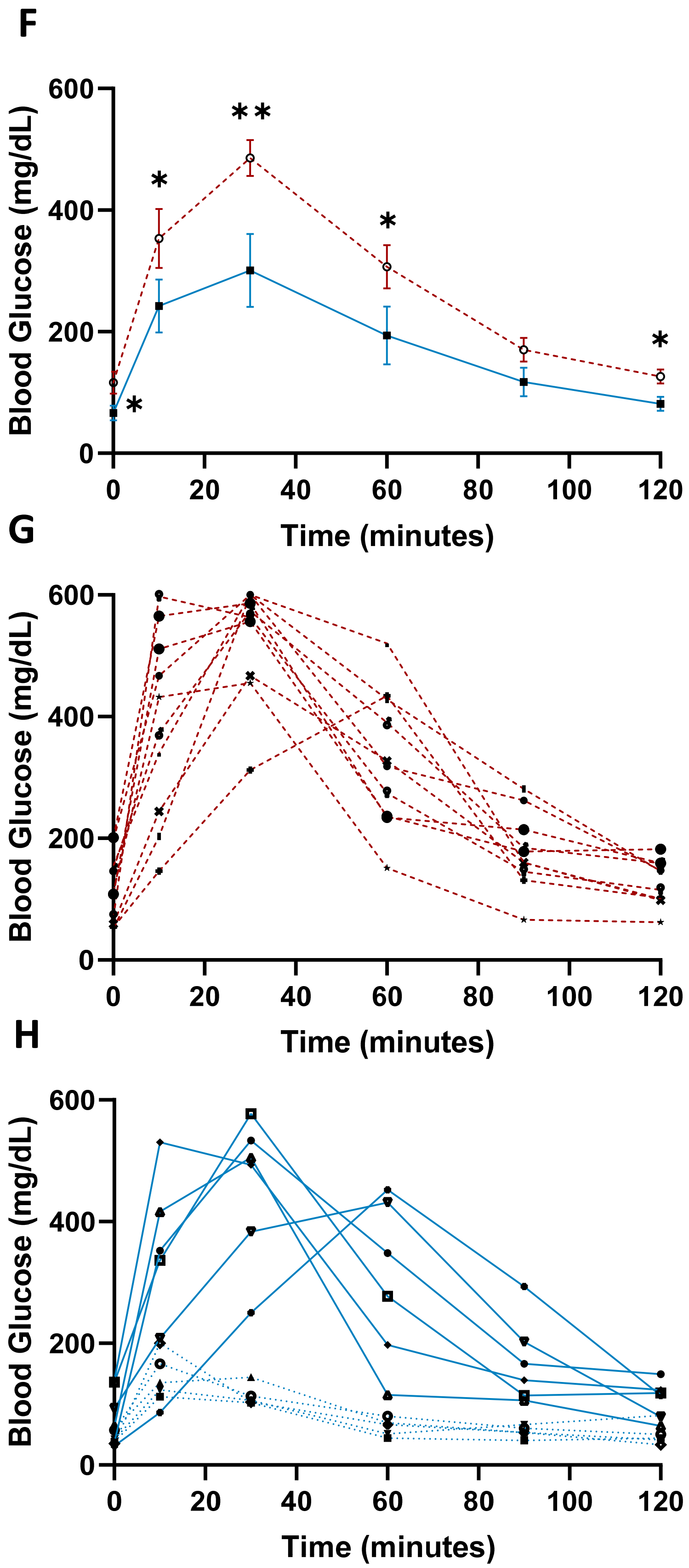
F
G
H
Blood Glucose (mg/dL)
Time (minutes)
600
400
200
0
20
40
60
80
100
120
*
**

I

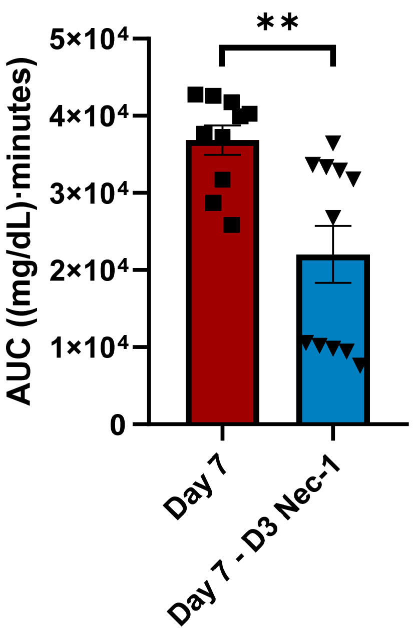

Supplementary Figure S4. Long-term metabolic follow-up of diabetic athymic nude mice after islet transplantation with PPIs cultured for 7 days in control media (n=10, dashed red line) or media supplemented with Nec-1 on day 3 of tissue culture (n=11, solid blue line). Athymic nude mice were rendered diabetic by an intraperitoneal streptozotocin injection, transplanted with 5000 IEQs of PPIs under the kidney capsule, and followed for 22 weeks. A) Average weekly non-fasting blood glucose measurements from 0 to 22 weeks after islet transplantation. B) Weekly non-fasting blood glucose measurements from 0 to 22 weeks of mice transplanted with PPIs cultured for 7 days in control media. C) Weekly non-fasting blood glucose measurements from 0 to 22 weeks of mice transplanted with PPIs cultured for 7 days in media supplemented with Nec-1 on day 3 of tissue culture. D) Average weekly body weight measurements from 0 to 22 weeks after islet transplantation. E) Porcine insulin measurements in serum of mice at 22 weeks after islet transplantation. F) Average blood glucose measurements during an OGTT (3 mg/kg) at 22 weeks after islet transplantation. G) Blood glucose measurements during an OGTT (3 mg/kg) of mice transplanted with PPIs cultured for 7 days in control media at 22 weeks after islet transplantation. H) Blood glucose measurements during an OGTT (3 mg/kg) of mice transplanted with PPIs cultured for 7 days in media supplemented with Nec-1 on day 3 of tissue culture at 22 weeks after islet transplantation. I) Glucose clearance after an OGTT (3 mg/kg) at 22 weeks post-transplantation expressed as AUC. Downward arrows indicate time of implantation of an insulin pellet. Upward arrows indicate time of nephrectomy of graft-bearing kidneys. Dotted black lines indicate the lower limit of the assay range (2.3 mU/L). *p<.05. **p<.01. Data expressed as mean ± SEM.

Supplementary Methods – Islet Viability

To assess islet viability, 100 IEQs were stained in Calcein AM (CalAM, 1:20; cat# C1430, Invitrogen) and propidium iodide (PI, 1:20; cat# P3566, Invitrogen) for 30 minutes and evaluated on a microplate reader (Infinite F200, Tecan)[15].

Supplementary Methods – Oxygen Consumption Rate (OCR)

200 IEQs were loaded into a titanium chamber connected to a fiber optic oxygen sensor (cat# FOL/C2T175P, Instech Laboratories) and filled with 37°C serum-free RPMI-1640 media[23]. The OCR was calculated as the linear decrease in the partial pressure of oxygen inside the chamber and normalized to the DNA content[25].
