# Supplementary material for: Necrostatin-1 Supplementation to Islet Tissue Culture Enhances the In-Vitro Development and Graft Function of Young Porcine Islets
Source: Int J Mol Sci. 2021 Aug 4;22(16):8367. doi: 10.3390/ijms22168367 (PMC8394857; doi:10.3390/ijms22168367)
Supplement: Supplementary file 1 [file ijms-22-08367-s001.zip › Nec-1 D3 vs. D0 - Supplementary Figures.pptx]

## Slide 1
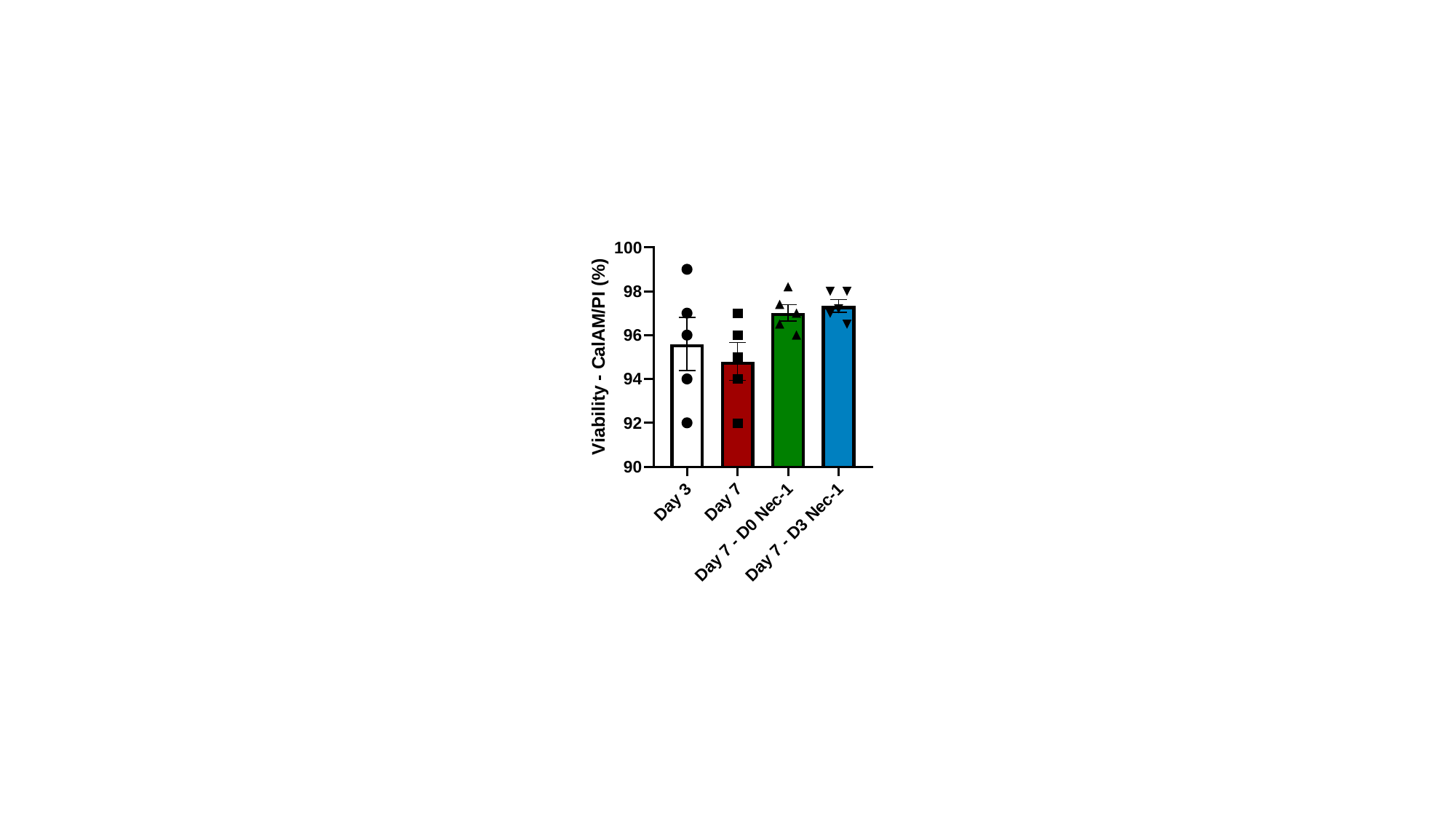

## Slide 2
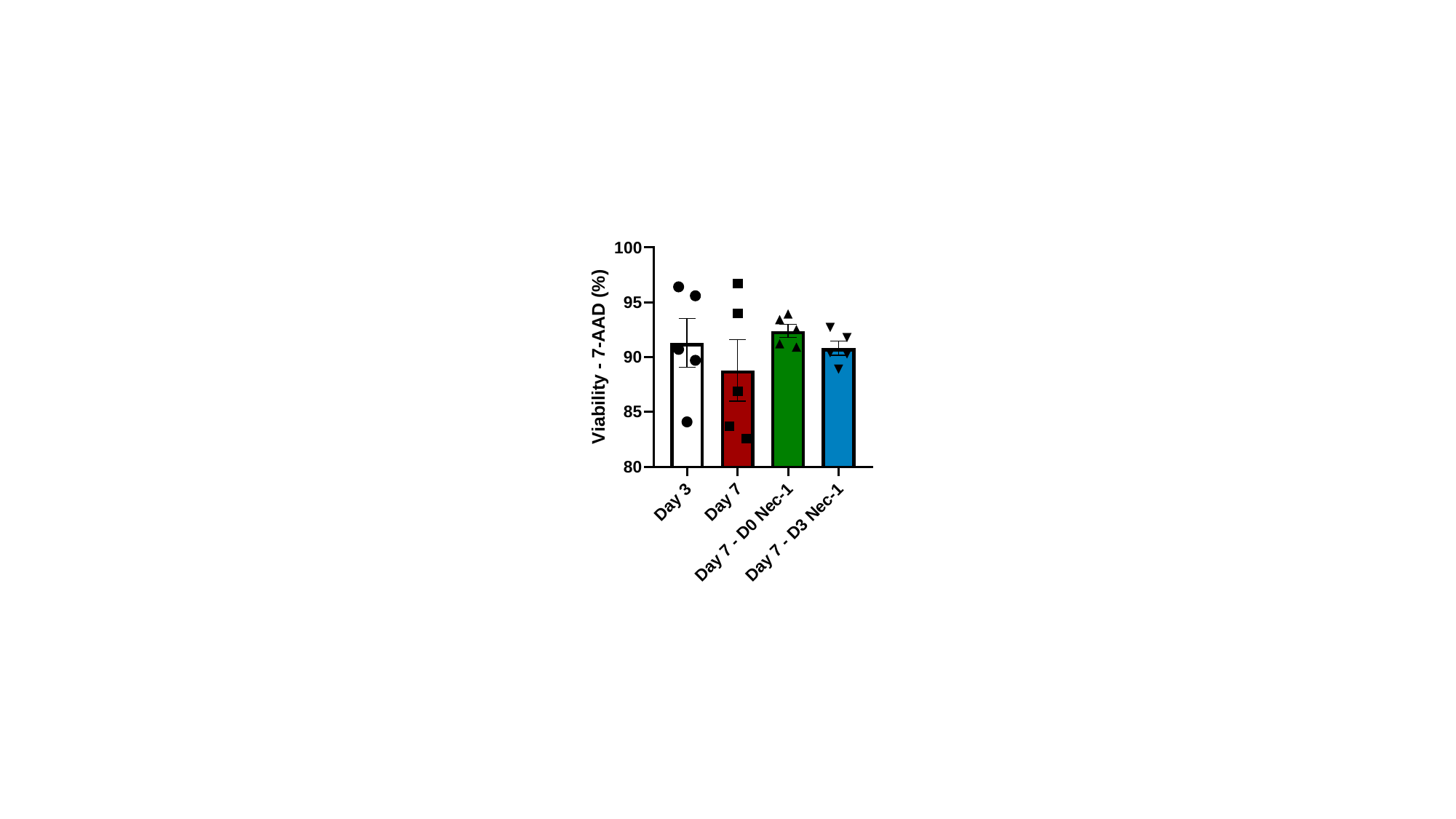

## Slide 3
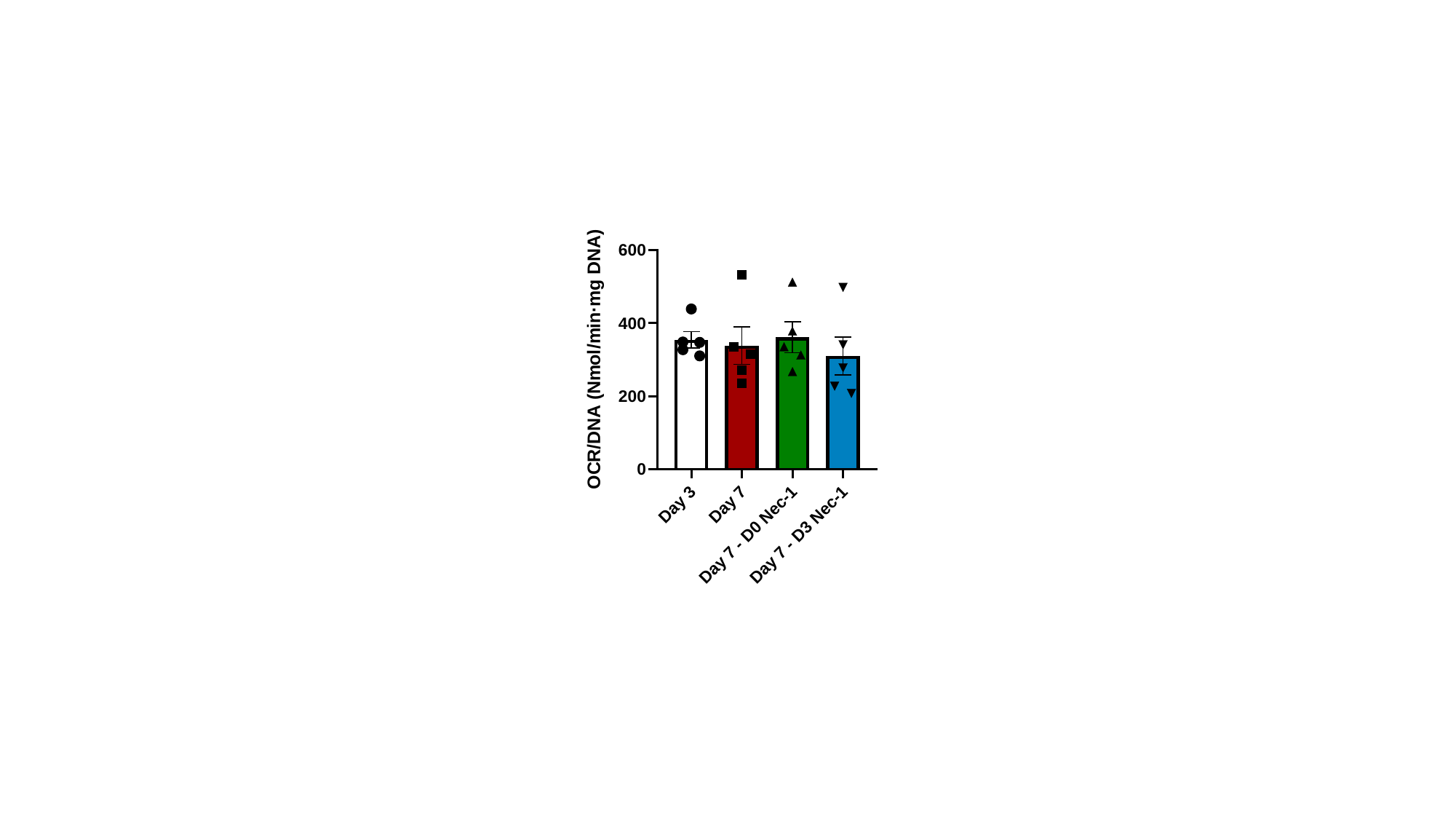

## Slide 4
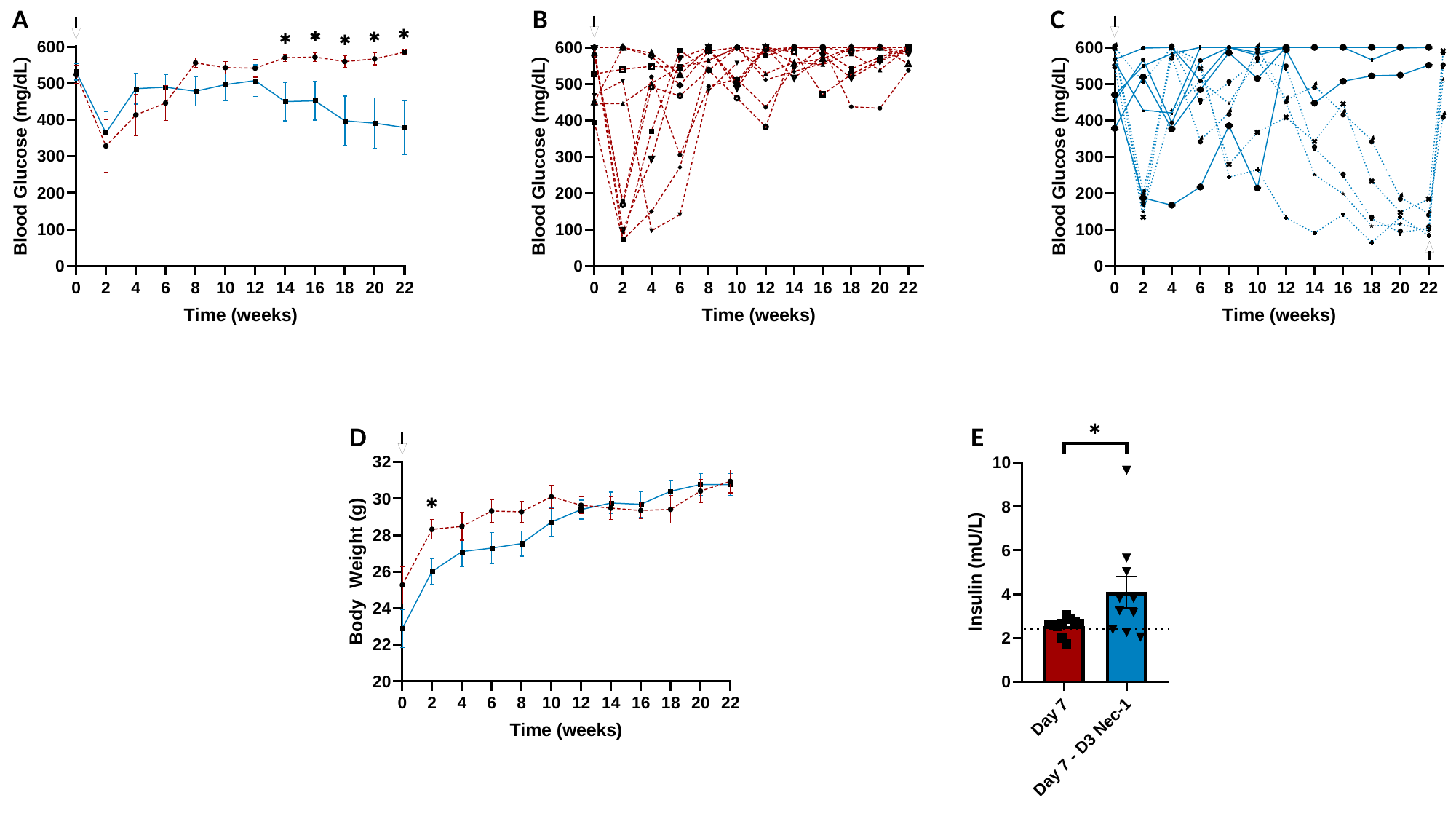

A
B
C
D
E

## Slide 5
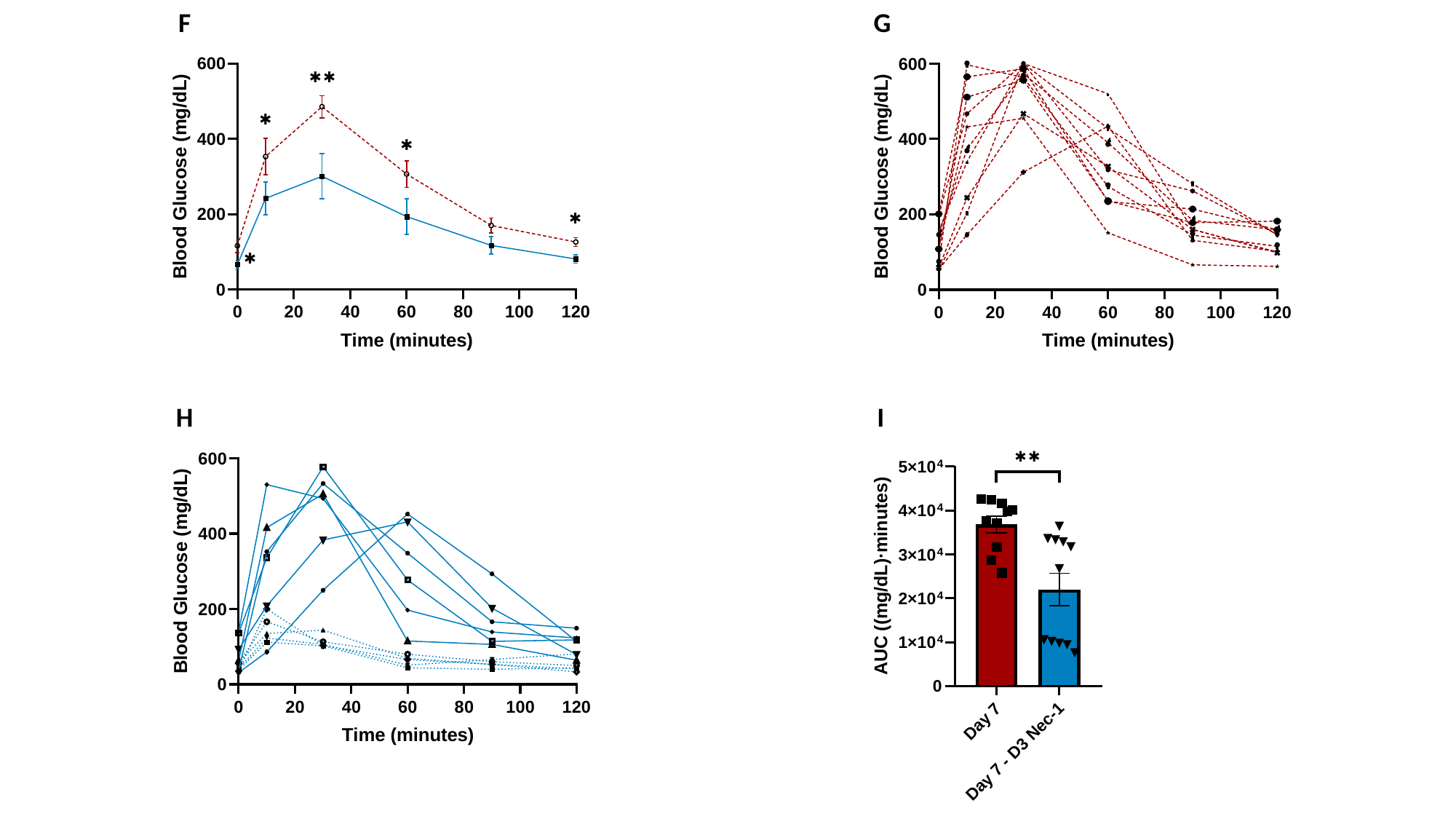

F
G
H
I

## Slide 6
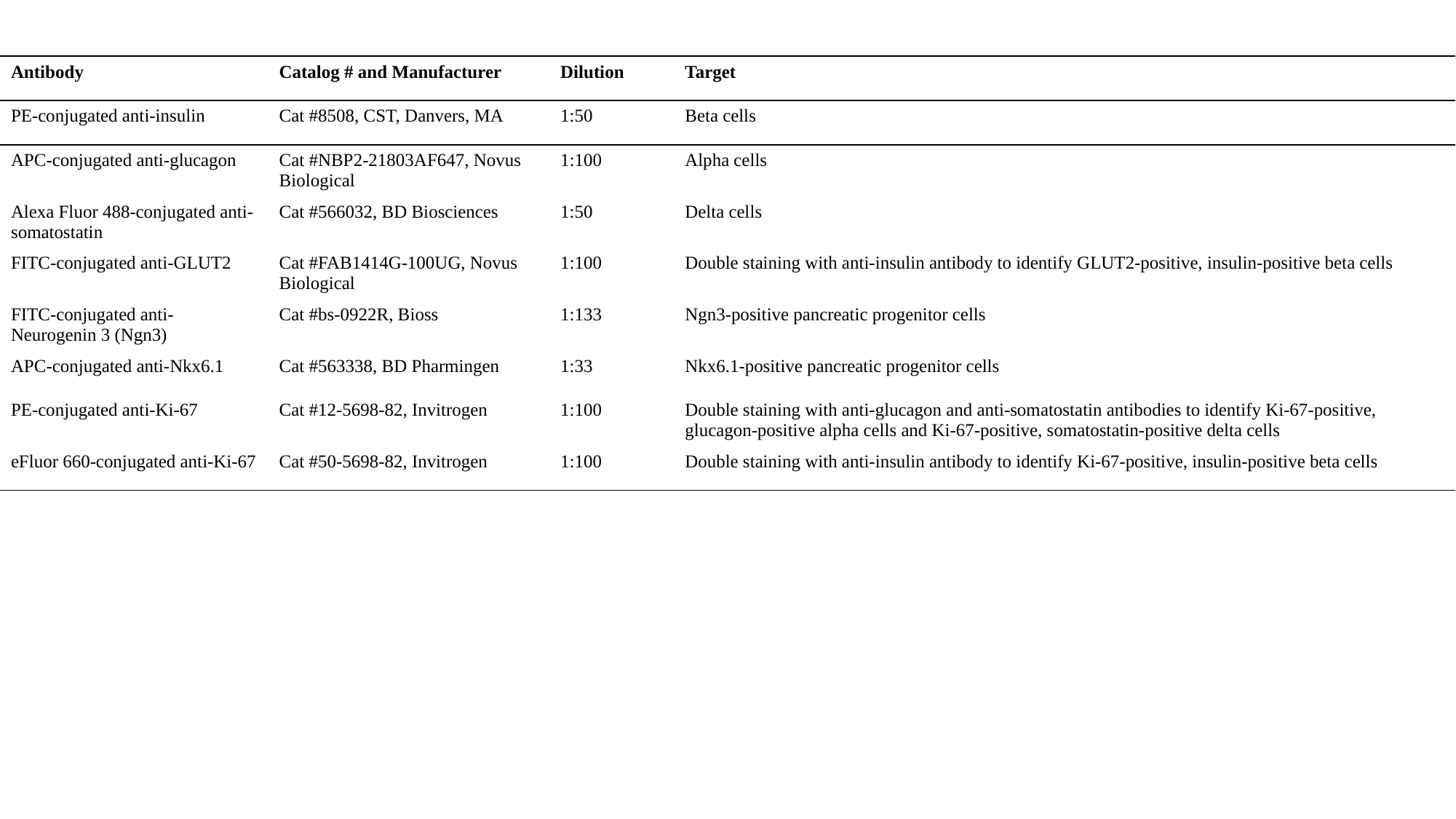

| Antibody | Catalog # and Manufacturer | Dilution | Target |
| --- | --- | --- | --- |
| PE-conjugated anti-insulin | Cat #8508, CST, Danvers, MA | 1:50 | Beta cells |
| APC-conjugated anti-glucagon | Cat #NBP2-21803AF647, Novus Biological | 1:100 | Alpha cells |
| Alexa Fluor 488-conjugated anti-somatostatin | Cat #566032, BD Biosciences | 1:50 | Delta cells |
| FITC-conjugated anti-GLUT2 | Cat #FAB1414G-100UG, Novus Biological | 1:100 | Double staining with anti-insulin antibody to identify GLUT2-positive, insulin-positive beta cells |
| FITC-conjugated anti-Neurogenin 3 (Ngn3) | Cat #bs-0922R, Bioss | 1:133 | Ngn3-positive pancreatic progenitor cells |
| APC-conjugated anti-Nkx6.1 | Cat #563338, BD Pharmingen | 1:33 | Nkx6.1-positive pancreatic progenitor cells |
| PE-conjugated anti-Ki-67 | Cat #12-5698-82, Invitrogen | 1:100 | Double staining with anti-glucagon and anti-somatostatin antibodies to identify Ki-67-positive, glucagon-positive alpha cells and Ki-67-positive, somatostatin-positive delta cells |
| eFluor 660-conjugated anti-Ki-67 | Cat #50-5698-82, Invitrogen | 1:100 | Double staining with anti-insulin antibody to identify Ki-67-positive, insulin-positive beta cells |
